# Downregulation of KLF10 contributes to the regeneration of survived renal tubular cells in cisplatin-induced acute kidney injury via ZBTB7A-KLF10-PTEN axis

**DOI:** 10.1038/s41420-023-01381-6

**Published:** 2023-03-06

**Authors:** Yang Zhang, Siyu Bao, Daxi Wang, Wei Lu, Sujuan Xu, Weiran Zhou, Xiaoyan Wang, Xialian Xu, Xiaoqiang Ding, Shuan Zhao

**Affiliations:** 1 Department of Nephrology, Zhongshan Hospital, Fudan University, Shanghai, China; 2 Shanghai Medical Center of Kidney Disease, Shanghai, China; 3 Kidney and Dialysis Institute of Shanghai, Shanghai, China; 4 Kidney and Blood Purification Key Laboratory of Shanghai, Shanghai, China

**Keywords:** Acute kidney injury, Cell division

## Abstract

Acute kidney injury (AKI) is a common clinical dysfunction with complicated pathophysiology and limited therapeutic methods. Renal tubular injury and the following regeneration process play a vital role in the course of AKI, but the underlining molecular mechanism remains unclear. In this study, network-based analysis of online transcriptional data of human kidney found that KLF10 was closely related to renal function, tubular injury and regeneration in various renal diseases. Three classical mouse models confirmed the downregulation of KLF10 in AKI and its correlation with tubular regeneration and AKI outcome. The 3D renal tubular model in vitro and fluorescent visualization system of cellular proliferation were constructed to show that KLF10 declined in survived cells but increased during tubular formation or conquering proliferative impediment. Furthermore, overexpression of KLF10 significantly inhibited, whereas knockdown of KLF10 extremely promoted the capacity of proliferation, injury repairing and lumen-formation of renal tubular cells. In mechanism, PTEN/AKT pathway were validated as the downstream of KLF10 and participated in its regulation of tubular regeneration. By adopting proteomic mass spectrum and dual-luciferase reporter assay, ZBTB7A were found to be the upstream transcription factor of KLF10. Our findings suggest that downregulation of KLF10 positively contributed to tubular regeneration in cisplatin induced acute kidney injury via ZBTB7A-KLF10-PTEN axis, which gives insight into the novel therapeutic and diagnostical target of AKI.

## Introduction

Acute kidney injury, a syndrome manifested as a rapid decline of renal function, is a common organ dysfunction caused by chemotherapy, surgery, sepsis and etc. According to a meta-analysis of world incidence of AKI, 1 in 5 adults and 1 in 3 children worldwide experience AKI during a hospital episode of care [1]. Nowadays, there are limited diagnostic or therapeutic options for early-stage AKI, thereby molecular mechanisms underlying the progression and recovery of AKI need excavating urgently. Cisplatin received FDA approval for the treatment of kinds of cancer in 1978, which still prevails in the current clinical treatment [2], such as cancers of breast, cervical, esophageal, bladder, small cell lung, and testicular [3–7]. However, AKI occurs frequently even with low-dose cisplatin administration [8], especially the acute tubular necrosis (ATN) [9]. Reversely, Pathophysiology of cisplatin-induced AKI remains shrouded in mystery.

Renal tubules are the most susceptible part of the kidney, especially in cisplatin-induced AKI [10]. AKI causes the injured tubular cells to shed and the remaining tubule begins to repair [11]. However, the mechanism of tubular regeneration still remains obscure. Several studies have found that resident survived tubular cells instead of circulatory stem cells play a significant role in repairing during AKI [12–14], either localized at the urinary pole of Bowman’s capsule or segment-specific and localized separately [11, 15–18]. CD133, CD24 and Vcam1 positive cells tended to be resource proliferative cells responsible for regeneration [19–21]. As activators, molecules such as Pax2, Sox9 and Foxm1 [14, 21, 22] and pathway like EGFR, Wnt-β catenin and Hippo signaling also got involved in the proliferation progress [23–25]. However, the inhibitors in tubular regeneration in AKI are lack of study, for which the subtle cellular proliferation progress cannot be fully understood. Thus, investigating the mechanisms of resident cell-cycling reentry and cease during AKI progression and repairing is of great significance.

Krüppel-like factor 10 (KLF10) is a well-known tumor suppressor of KLFs family because of its inhibitory effect on cell proliferation, and has become a research target for lung cancer, pancreatic cancer and liver cancer [26], which is first discovered as TGFβ inducible early gene 1 in osteoblasts [27]. Moreover, several studies have shown that KLF10 is also involved in the pathophysiology of various acute tissue injury diseases by inhibiting the proliferation of injured cells. In cerebral ischemia-reperfusion injury, down-regulation of KLF10 can inhibit N-myc/PTEN signaling pathway and promote the proliferation and repair of brain nerve cells [28]. In lower extremity ischemia-reperfusion injury, down-regulation of KLF10 can inhibit TGFβ/Smad pathway, alleviate cell cycle arrest caused by injury, and promote proliferation and repair of lower extremity vascular endothelial cells [29]. In the field of nephrology, KLF10 was reported to aggravate diabetic podocyte dysfunction while its role in renal tubular cell remains unclear [30]. Apart from that, it is lack of exploration in kidney, especially in AKI. Therefore, the effect of KLF10 on the proliferation of renal tubular epithelial cells in AKI is worth studying.

In our current work, we used transcriptome combined with clinical data to uncover the relationship between KLF10 and renal function or tubular proliferation. Through not only adoption of classical AKI mouse models in vivo but also construction of 3D renal tubular model in vitro, the role of KLF10 in injury repairing, tubular proliferation and lumen-formation during the course of AKI was further validated. Mechanistically, the up- and down-stream of KLF10 in regulating tubular proliferation were also investigated. Collectively, these findings reveal that KLF10 hinders the regeneration of renal tubules in cisplatin-induced AKI via ZBTB7A-KLF10-PTEN axis, which provides a potential approach for the diagnosis and treatment of AKI.

## Results

### The expression of KLF10 in kidney was highly correlated with renal function, renal tubular proliferation and various renal diseases in human datasets

**Fig. 1:**
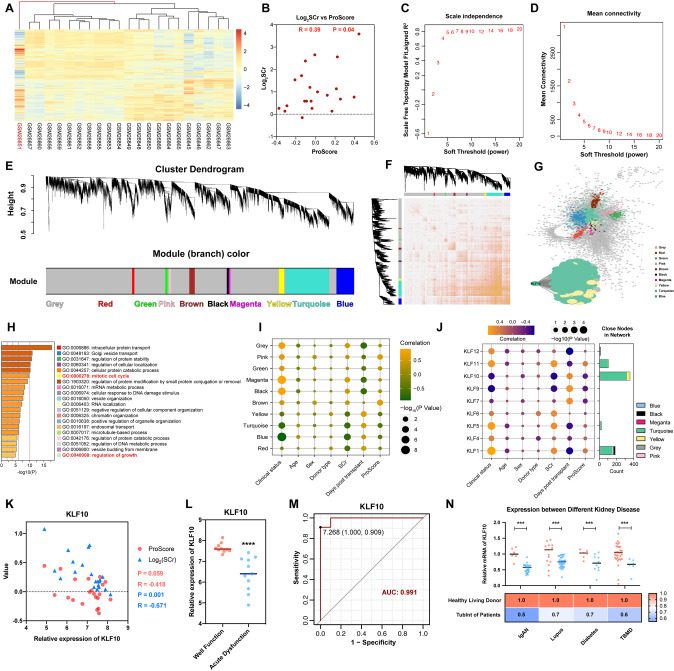
Construction of weighted gene co-expression network and clinical and functional analysis of KLF10. **A** Heatmap with hierarchical clustering dendrogram of kidney samples in GSE1563. **B** Negative correlation between Epithelial Proliferation Score (ProScore) and Log_2_ (Serum Creatinine). **C** Analysis of appropriate soft-thresholding powers for WGCNA. **D** Average network connectivity under weighting coefficients of WGCNA. **E** Clustering dendrograms of genes in 21 samples, from which 10 coexpression modules were constructed with different colors. **F** The Heatmap plot depicts the TOM among all genes in the analysis, which shows the interactions among co-expression modules. The stronger intensity of orange indicates greater overlaps. **G** Interaction network analysis based on WGCNA and network of KLF10 with its closely interacted genes. Each dot is color-coded by its corresponding module. **H** Histogram of functional enrichment analysis of genes closely interacted with KLF10. **I** Bubble plot of the relationship between the coexpression module and clinical data. The size shows the corresponding -log_10_ (*P* value) and the color represents the correlation coefficient. **J** Bubble plot of the relationship between the KLF family and clinical data with histogram displaying the number of closely interacted genes with each KLF. The size shows the corresponding -log_10_ (*P* value) and the color represents the correlation coefficient. **K** Negative correlation between KLF10 and ProScore/Log_2_ (Serum Creatinine). **L** Scatter plot of relative expression of KLF10 in well function group versus acute dysfunction group. **M** ROC curve with AUC value of KLF10. **N** Scatter plot of tubulointerstitial relative expression of KLF10 in control groups versus renal disease groups. ***p < 0.001 and ****p < 0.0001 vs control group at the same experimental conditions.

In order to excavate the clinical meaning and biological function of KLF10, we analyzed human renal transcriptomic data of GSE1563 [31]. After outlier analysis (Fig. 1A), well-functioning transplants with no clinical evidence of rejection (clinical status: 1, *n* = 10), transplants with acute renal dysfunction without rejection (clinical status: 2, *n* = 5), and kidneys undergoing acute rejection (clinical status: 3, *n* = 6, 1 outlier) were included in the following analysis. To evaluate the tubular proliferation level of each sample, gene set in corresponding GO terms were taken into the gene set variation analysis labeled with Epithelial Proliferation Score (ProScore, Table 1). (Considering that the kidney from healthy living donor did not conquer transplant attack or related drug usage which was far more different from transplant kidney and affected the quality of analysis, we used well-functioning transplants as the control instead.) Interestingly, the ProScore was positively correlated to Log_2_SCr in the scope of the dataset, which confirmed that the renal tubules prone to proliferate as kidney injury progressed (Fig. 1B). The baseline characteristics of the patient were depicted in the Table 2. The genes with NA value or duplicate values except the maximum one was removed and 21 samples and 9155 genes were obtained finally. The soft thresholding power was set as 7.0 with correlation over 0.8 after careful consideration (Fig. 1C, D). Then the gene expression profiles could be transformed into the adjacency matrix, TOM, and dissTOM and 10 co-expression modules were constructed subsequently (Fig. 1E). Moreover, dissTOMplot showed that the modules were relatively independent of each other suggesting efficient clustering (Fig. 1F). By adopting Cytoscape, we transferred the dissTOM into protein-to-protein interaction network based on the correlation strength among each genes. The KLF10 with its closely related genes was finally obtained (Fig. 1G), which showed that both KLF10 and its neighboring genes belonged mainly to the turquoise module.

**Table 1:** Selection of GO terms of Epithelial Proliferation Score (ProScore).

Screening condition	AND/NOT	Positive regulation of epithelial cell proliferation (PRECP)	Negative regulation of epithelial cell proliferation (NRECP)
GO Term	AND	GO:0050679	GO:0050680
Organism	AND	Homo sapiens	Homo sapiens
Type	AND	protein	protein
Evidence	AND	experimental evidence	experimental evidence
GO class	NOT	positive regulation of endothelial cell proliferation	negative regulation of endothelial cell proliferation
		positive regulation of vascular endothelial cell proliferation	negative regulation of blood vessel endothelial cell proliferation involved in sprouting angiogenesis
		positive regulation of blood vessel endothelial cell proliferation involved in sprouting angiogenesis	negative regulation of vascular endothelial cell proliferation
Number of Genes for Gene Set Variation Analysis (GSVA)		55	33
Epithelial Proliferation Score (ProScore)		= PRECP − NRECP

**Table 2:** Baseline characteristics of the patient taken into WGCNA.

	Well functioning graft	Acutely dysfunctional graft without rejection	Graft with acute rejection	*P*-value
*n*	10	5	6	-
Age (mean (SD))	44.70 (12.77)	42.20 (15.77)	32.83 (13.33)	0.259
Male (%)	7 (70.0)	4 (80.0)	4 (66.7)	0.88
Cadaveric Donor (%)	5 (50.0)	1 (20.0)	5 (83.3)	0.109
SCr (mean (SD))	1.32 (0.27)	3.76 (1.94)	4.42 (4.03)	0.0001
Days post transplant (mean (SD))	769.90 (105.85)	143.20 (184.32)	430.50 (519.88)	0.031
ProScore (mean (SD))	−0.08 (0.21)	0.02 (0.22)	0.10 (0.24)	0.363

Functional enrichment analysis of the KLF10 cluster above indicated that KLF10 might participate in the ‘*mitotic cell cycle*’ and ‘*regulation of growth*’ (Fig. 1H). Then, the correlation coefficients for the modules/KLF family and the clinical data were calculated to identify how each module or gene was related to each clinical trait. It was obvious that both KLF10 and its belonging turquoise module were negatively related to clinical status, serum creatinine and regeneration of renal tubules (Fig. 1I–K). KLF10 also showed the most closely related genes among the KLF family which illustrated that KLF10 might played the greatest role in AKI versus the other KLF members (Fig. 1J). Correspondence to the results above, KLF10 decreased significantly in acute dysfunction group and ROC curve with AUC value over 0.9 showed high specificity and sensitivity of KLF10 to AKI (Fig. 1L, M). Furthermore, renal tubulointerstitial RNA-seq data of other kidney diseases from Nephroseq database also displayed significant downregulation of KLF10 (Fig. 1N), which validated the vital role of KLF10 in injury renal tubule. Above all, the level of KLF10 in kidney was clinically correlated to renal function and renal tubular regeneration.

### KLF10 dramatically decreased in AKI mouse models and in 3D renal tubular injury model, correlated tightly with the proliferative states of renal epithelial cells

**Fig. 2:**
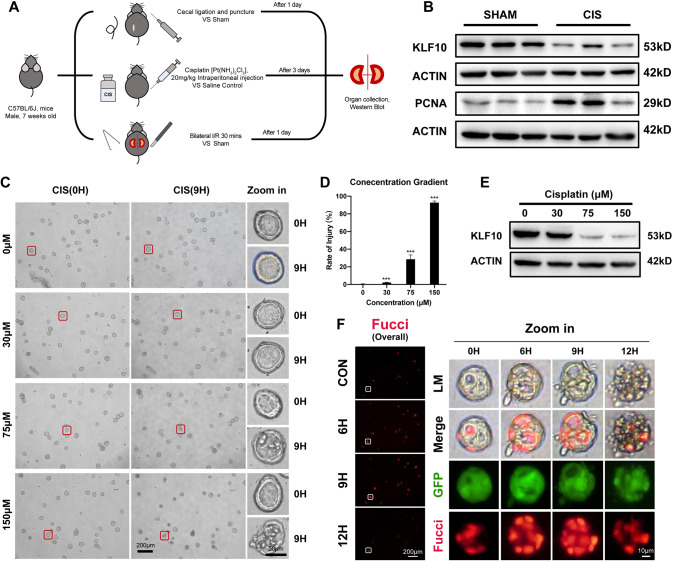
KLF10 was down-regulated in multiple AKI mouse models and 3D renal tubule injury models. **A** Flowchart detailing bilateral IR or CLP surgery and cisplatin treatment regime in C57BL/6 J mice. **B** Representative immunoblot analysis of KLF10 and PCNA in kidney tissues from cisplatin-induced AKI mouse model. ACTIN served as the standard. *n* = 6 per group. **C**, **D** The morphology and injury rate of 3D renal tubular model treated with cisplatin of different concentration. **E** Representative immunoblot analysis of KLF10 in cisplatin treated 3D renal tubular model. ACTIN served as the standard. **F** Fluorescence images of 3D tubular cisplatin-induced AKI model (75 μM) at different time points reflecting the injury degree (LM - light microscope and GFP) and proliferative activity (Fucci-overall, Merge and Fucci). ***p < 0.001 vs control group at the same experimental conditions.

Relevance between downregulation of KLF10 and AKI was further investigated in three classical AKI mouse models. Mice were treated with 30 min IR or 20 mg/kg cisplatin intraperitoneally administered or cecum ligation and puncture (CLP) and then sacrificed at corresponding time point respectively (Fig. 2A). All three models showed significant decrease in renal expression of KLF10 versus control group. Moreover, proliferation marker PCNA of IR group and cisplatin group and CCNB1 of CLP group increased, suggesting the occurrence of cellular proliferation in AKI (Fig. 2B and Supplementary Figs. 2A, 3A).

To validate the in vivo results, 3D renal tubular injury in vitro model was constructed. We observed time- and dose-dependent changes in tubular morphology, which were highly similar to tubular changes of AKI in vivo, including cell shedding, apoptosis and necrosis (Fig. 2C, D and Supplementary Figs. 1A, B). Downregulation of KLF10 was also found in this model (Fig. 2E). The degrees of tubular injury and proliferation at different time points were then observed (Fig. 2F). The proliferation was significantly activated as the damage progressed (Fig. 2F - LM/GFP) and peaked at 9 h (Fig. 2F - Merge/Fucci/Overall), which was consistent with the changes of KLF10 in vitro/in vivo and PCNA in vivo. However, the proliferation was suppressed at 12 h because the structure of tubular organoid was lost mostly and tubular cell death occurred the most. Thus, KLF10 might get involved in the tubular injury and tubular proliferation of cisplatin-induced AKI.

**Fig. 3:**
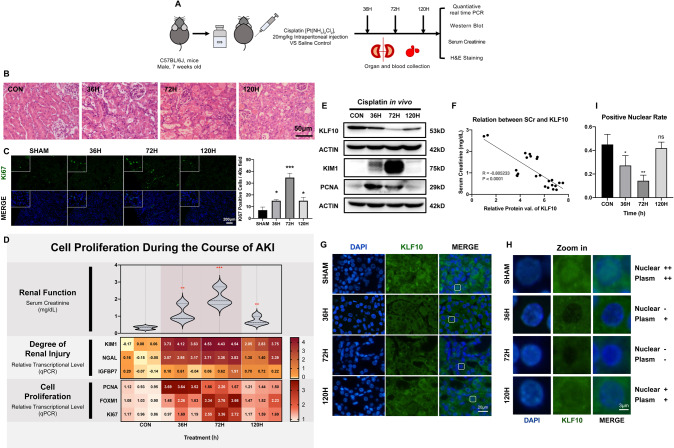
Downregulation of KLF10 was correlated with cell proliferation and the outcome of cisplatin-induced AKI in vivo. **A** Flowchart detailing cisplatin treatment regime in C57BL/6 J mice. *n* = 6 per group. **B** Representative H&E staining image of kidney sections from the model. **C** Representative immunofluorescence staining image of Ki67 in the model and counts of positive cells per 40x field (objective lens). **D** Renal dysfunction was determined in cisplatin (20 mg/kg, ip) treated mouse model. Serum creatinine (SCr) was measured in sera. Degree of renal injury was determined in the model through relative mRNA levels of KIM1, NGAL and IGFBP7 in mouse kidney tissues. Degree of renal cellular proliferation was determined in the model through relative mRNA levels of PCNA, FOXM1 and Ki67 in mice kidney tissues. **E** Representative immunoblot analysis of KLF10, KIM1 and PCNA in the model. ACTIN served as the standard. **F** Regression analysis was undertaken to determine correlation between relative protein level of KLF10 in mice kidney tissues and SCr. Relative protein level of KLF10 was examined and normalized by Fiji. **G**–**I** Representative immunofluorescence staining image and rate of positive nuclear of KLF10 in the model. *p < 0.05, **p < 0.01, ***p < 0.001, ns no significantly difference vs control group at the same experimental conditions.

Given that KLF10 got involved in the tubular injury process of AKI, we further explored the change of KLF10 as the AKI recovered. A dose of 20 mg/kg cisplatin was intraperitoneally administered to C57BL/6 J mice with kidney harvested 36, 72 and 120 h later (Fig. 3A). H&E staining also showed obvious morphological changes on day 3 including loss of tubular brush, vacuolar degeneration and acute tubular necrosis, which got milder on day 5 (Fig. 3B). SCr, KIM1, and NGAL ascended the most significantly at 72 h and descending at 120 h, suggesting that the renal function got worst on day 3 and moderately recovered on day 5 (Fig. 3D). Interestingly, levels of cell proliferation markers PCNA, FOXM1 and Ki67 as well as KLF10 developed the same changing trend (Fig. 3C–E). Moreover, KLF10 was observed in plasm and nuclei of renal tubules and both levels were reduced and then regained as AKI progressed and then recovered (Fig. 3G–I). Regression analysis was then undertaken based on KLF10 protein level and SCr/blood urea nitrogen (BUN)/KIM1, for further validation of the relevance between KLF10 and renal function and injury (Fig. 3F and Supplementary Fig. 4). The decrease and recovery of KLF10 could be observed in IR- and CLP-induced mouse model as well (Supplementary Figs. 2B, 3B).

**Fig. 4:**
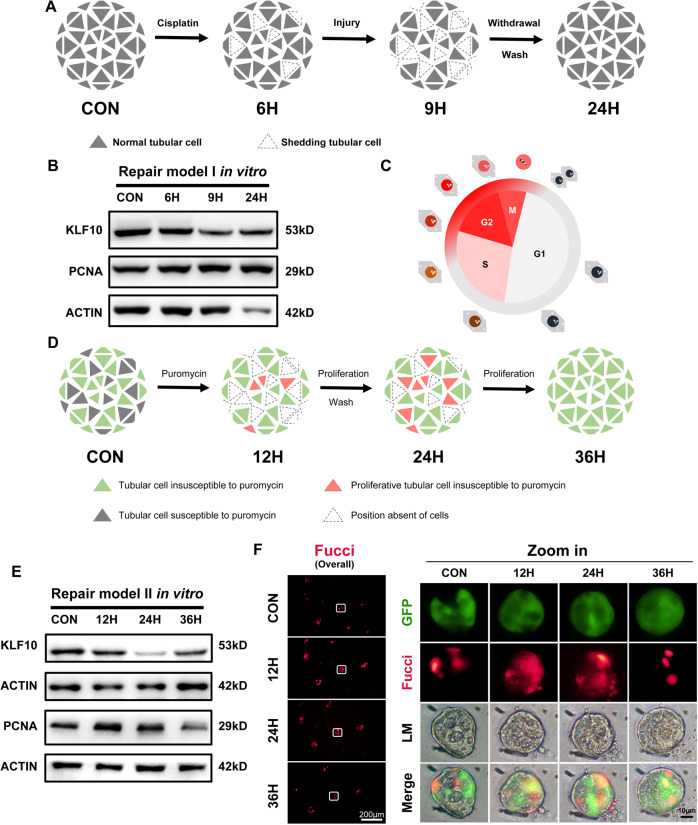
Downregulation of KLF10 in survived tubular cells was reversed as the integrity of injury 3D renal tubules recovered in vitro. **A** Diagram detailing cisplatin treatment regime in 3D renal tubular model. **B** Representative immunoblot analysis of KLF10 and PCNA in model of Fig. 4A. ACTIN served as the standard. **C** Diagram detailing the change in flurescent color of Fucci-cell during different stages of cell cycle. **D** Diagram detailing puromycin treatment regime in 3D renal tubular model. **E** Representative immunoblot analysis of KLF10 and PCNA in model of Fig. 4D. ACTIN served as the standard. **F** Fluorescence images of 3D survived tubular repair model according to the treatment in Fig. 4D, reflecting the forming integrity of survived tubular cells (LM and GFP) and proliferative activity (Fucci-overall, Merge and Fucci).

To specified KLF10 changes in renal tubular cells further, 3D renal tubular injury model in vitro was treated with 75 μM cisplatin for 6 h or 9 h and then cultured in normal condition without cisplatin for another 15 h to recover (Fig. 4A). The KLF10 decreased after 9 h treatment and rose up after cisplatin withdrawal for 15 h (Fig. 4B). Fucci sensor cellular system [32] was constructed to visualize cell cycle progression, which showed red fluorescence (mCherry) in G2/M cells (Fig. 4C). To dissect the change of KLF10 in the survived cells from that in the apoptotic or necrotic one, Fucci renal tubule cell (without green fluorescence) and puromycin-resistant Fucci renal tubule cell (with green fluorescence) were mixed at the ratio of 1:1 and then cultured in matrix for 6 days to form 3D renal tubule. Puromycin was added for 12 h to induce apoptosis of the sensitive cells, the rest continued to be cultured for another 12 h or 24 h (Fig. 4D). The density of red fluorescence raised dramatically at 12 h and 24 h and then declined at 36 h (Fig. 4F Fucci fluorescence) as the tubule fully repaired (Fig. 4F GFP fluorescence), which was confirmed further by the change of PCNA (Fig. 4E). Consistent with results in vivo and in vitro above, the expression pattern of KLF10 was opposite to that of PCNA (Fig. 4E). Therefore, the change of KLF10 expression level reflects the injury, healthy or recovery state of renal tubules throughout the course of AKI both in vivo and in vitro.

### KLF10 was an inhibitor of tubular cell proliferation in AKI

**Fig. 5:**
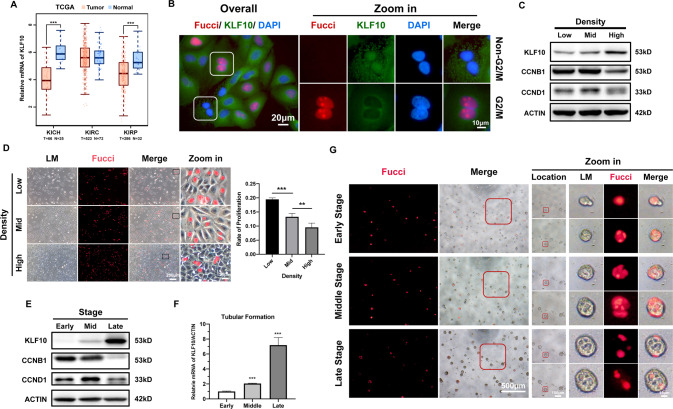
KLF10 increased when experiencing physical inhibition to proliferation or during the formation of 3D renal tubule model in vitro. **A** Datasets published on GEPIA were examined for KLF10 transcript expression between tumor tissue (T) and normal tissue (N). KICH, Kidney chromophobe; KIRC, Kidney renal clear cell carcinoma; KIRP, Kidney renal papillary cell carcinoma. **B** Representative immunofluorescence staining image of KLF10 and Fucci in 2D cultured renal tubular cells. **C** Representative immunoblot analysis of KLF10, CCNB1 and CCND1 in low-/middle-/high-density group. ACTIN served as the standard. **D** Representative fluorescence image of Fucci in low-/middle-/high-density group. Rate of proliferation was calculated via percentage of Fucci positive cells. **E** Representative immunoblot analysis of KLF10, CCNB1 and CCND1 in early/middle/late stages of tubular formation in 3D renal tubular model. **F** Relative mRNA levels of KLF10 in different stages of tubular formation in 3D renal tubular model were examined. **G** Representative fluorescence image of Fucci during different stages of tubular formation in 3D renal tubular model (early stage: day 1–2, middle stage: day 3–4, late stage: day 5–6). ns *p* ≥ 0.05, **p < 0.01, ***p < 0.001 vs control group at the same experimental conditions.

Validation of the relationship between KLF10 and cell proliferation was then carried out. Firstly, analysis of datasets published on GEPIA indicated that renal tumor tissue (KICH, KIRP) had much lower level of KLF10 than the normal tissue (Fig. 5A) [33]. Secondly, immunofluorescence staining also demonstrated that there was less KLF10 in tubular cells in G2/M phase versus those in non-G2/M phase, especially in the nuclei (Fig. 5B). Moreover, when cell proliferation was inhibited by physically contraction, an elevated expression of KLF10 was observed compared to low density group (Fig. 5C, D). In addition, we mimicked the regeneration pattern of normal renal tubule in vitro, upregulation of KLF10 was observed at the late stage of tubular formation concomitant with lower rate of proliferation through levels of CCNB1, CCND1 and Fucci fluorescence (Fig. 5E–G).

**Fig. 6:**
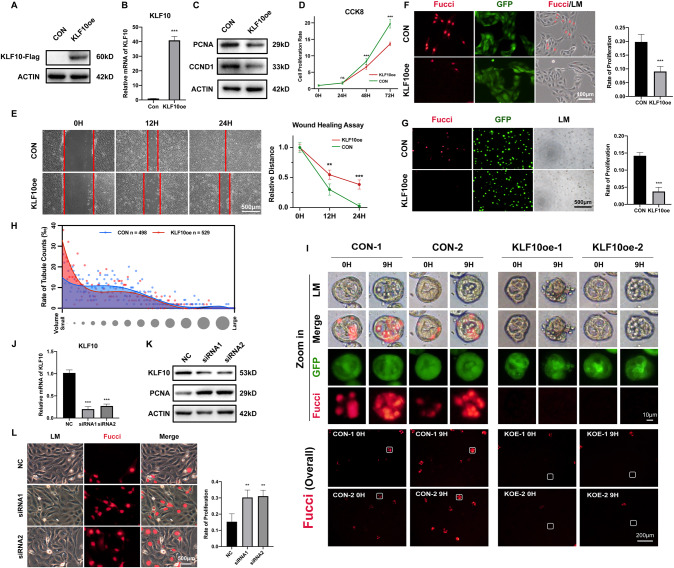
Overexpression or suppression of KLF10 affected the ability of proliferation, repair and lumen-formation of renal tubular cells. **A**, **B** Efficient overexpression of KLF10 was confirmed by immunoblot analysis and relative mRNA levels. **C** Representative immunoblot analysis of PCNA and CCND1 in KLF10 overexpressed group and control group. ACTIN served as the standard. **D** The cell viability of KLF10 overexpressed group and control group were measured by CCK-8 assay. **E** The ability to repair was measured by wound healing assay. **F**, **G** Representative fluorescence image of KLF10 overexpressed group and control group (2D/3D cultured). **H** Distribution of relative volume of tubule model was calculated and analyzed by Fiji. **I** Fluorescence images of 3D tubular cisplatin-induced AKI model (75 μM) for 9 h with or without overexpression of KLF10 reflecting the injury degree (LM and GFP) and proliferative activity (Fucci-overall, Merge and Fucci). **J** Relative mRNA levels of KLF10 in siRNA transfected group and control group were examined. **K** Representative immunoblot analysis of KLF10 and PCNA in siRNA transfected group and control group. ACTIN served as the standard. **L** Representative fluorescence image of Fucci in siRNA transfected group and control group. *p < 0.05, **p < 0.01, ***p < 0.001 vs control group at the same experimental conditions.

To gain more insight into the role of KLF10 in cell proliferation, tubular formation and repairing, overexpression of KLF10 was carried out in vitro (Fig. 6A, B). Expression of PCNA and CCND1 decreased in KLF10-overexpressed cells (Fig. 6C). Proliferation rate declined significantly as well in both 2D and 3D cultured in vitro model (Fig. 6F, G), which was validated further through CCK-8 assay (Fig. 6D). Overexpression of KLF10 also inhibited the ability to repair based on wound healing assay (Fig. 6E). We could also observe that KLF10-overexpressed 3D model showed larger rate of small tubules and less rate of large tubules (Fig. 6H). Then, we overexpressed KLF10 in 3D tubular organoid under cisplatin stimuli (Fig. 6I). As we expected, overexpression of KLF10 not only exacerbated tubular injury such as more shedding and death of tubular cells and loss of tubular structure (Fig. 6I - LM/GFP), but also inhibited the proliferation and activation of proliferation after cisplatin stimuli strongly (Fig. 6I - Merge/Fucci/Overall), validating the negative role of highly expressed KLF10 on cell proliferation under condition of cisplatin stimuli. Consistently, knockdown of KLF10 exerted converse effects on proliferation (Fig. 6J–L). Considering together, KLF10 got involved not only in the cell proliferation but also in the formation and repair of renal tubules in AKI.

### KLF10 impeded tubular regeneration via PTEN/AKT signaling

**Fig. 7:**
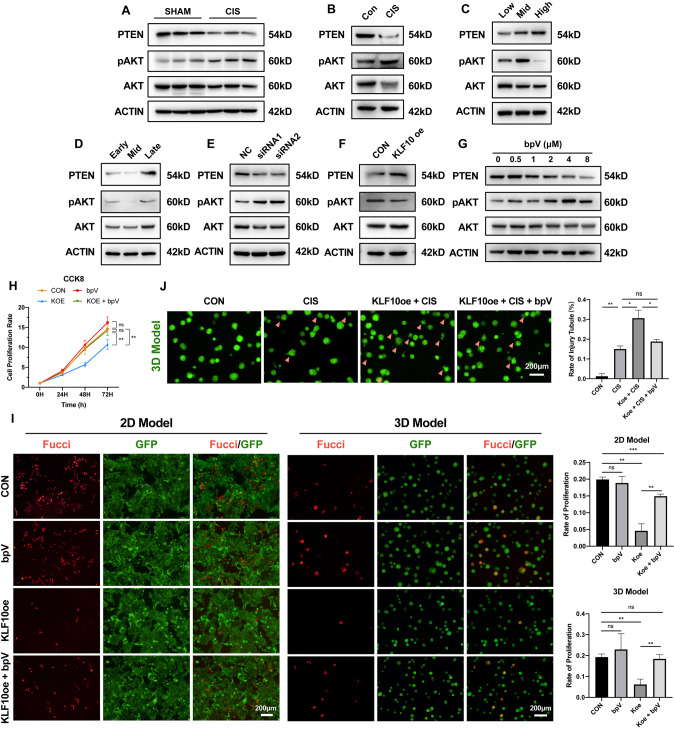
KLF10 inhibited tubular regeneration via PTEN/AKT pathway. **A** Representative immunoblot analysis of PTEN, pAKT and AKT in cisplatin-induced AKI group and control group. ACTIN served as the standard. *n* = 6 per group. **B** Representative immunoblot analysis of PTEN, pAKT and AKT in 3D AKI group and control group. ACTIN served as the standard. **C** Representative immunoblot analysis of PTEN, pAKT and AKT in low-/middle-/high-density group. ACTIN served as the standard. **D** Representative immunoblot analysis of PTEN, pAKT and AKT in early/middle/late stages of tubular formation in 3D renal tubular model. ACTIN served as the standard. **E** Representative immunoblot analysis of PTEN, pAKT and AKT in siRNA transfected group and control group. ACTIN served as the standard. **F** Representative immunoblot analysis of PTEN, pAKT and AKT in KLF10 overexpressed group and control group. ACTIN served as the standard. **G** Representative immunoblot analysis of PTEN, pAKT and AKT in bpV treatment group with different concentration. ACTIN served as the standard. **H** The cell viability in bpV (4 μM) treated/KLF10 overexpression/bpV (4 μM) treated + KLF10 overexpression group and control group were measured by CCK-8 assay. **I** Representative fluorescence image of Fucci in bpV (4 μM) treated/ KLF10 overexpression/ bpV (4 μM) treated + KLF10 overexpression group and control group (2D/3D cultured). **J** Representative image of 3D renal tubular model in cisplatin (75 μM) treated/cisplatin (75 μM) treated + KLF10 overexpression/cisplatin (75 μM) treated + KLF10 overexpression + bpV (4 μM) treated group and control group. ns *p* ≥ 0.05, *p < 0.05, **p < 0.01, ***p < 0.001 vs control group at the same experimental conditions.

To elucidate the mechanism by which KLF10 inhibits cell proliferation, we studied the PTEN/AKT pathway activation by KLF10 in AKI mouse models and renal tubular epithelial cells, because mounting evidence suggests that PTEN/AKT pathway has great negative effects on cellular proliferation and cerebral ischemia-reperfusion injury [28, 34, 35]. Decreasing PTEN with increasing phosphorylation of AKT was observed in IR-, cisplatin- and CLP-induced AKI mouse models (Fig. 7A and Supplementary Figs. 2C, 3C), which could be also examined in cisplatin treated 3D model in vitro (Fig. 7B). Consistent with the trend of KLF10 expression, PTEN elevated significantly not only in high-density cultured tubular cells but also in the late stage of tubular formation (Fig. 7C, D). Phosphorylation of AKT reduced obviously as well when tubular proliferation stagnated due to high-density. Interestingly, in the late stage of tubular formation, pAKT bumped up on the contrary which might be due to other potential mechanism in 3D cultured condition (Fig. 7D). Thus, PTEN/AKT also participated in the progression of AKI and tubular regeneration.

Next, in order to clarify the relationship between PTEN/AKT and KLF10, overexpression and suppression of KLF10 were carried out in vitro. Knockdown of KLF10 led to reduced PTEN and increased pAKT (Fig. 7E). On the other hand, PTEN increased evidently with decreased pAKT in KLF10 overexpressed group (Fig. 7F). Therefore, PTEN/AKT pathway was the downstream of KLF10.

Furthermore, we adopted bpV, an efficient PTEN-specific inhibitor [36], to regulate PTEN/AKT pathway in vitro. Treatment with bpV of 4 μM showed the weakest PTEN and the strongest pAKT in renal tubular cells (Fig. 7G), which was adopted in the following treatment. Through CCK8 assay, we found that inhibition of PTEN could reverse the negative effect of KLF10 overexpression on tubular proliferation (Fig. 7H), which was confirmed by the Fucci fluorescence in both 2D and 3D cultured model (Fig. 7I). Moreover, tubular injury deteriorated by overexpression of KLF10 while inhibition of PTEN alleviated the worsen condition (Fig. 7J). Overall, KLF10 impeded tubular regeneration to worsen renal injury via PTEN/AKT pathway.

### The inhibitive effect of KLF10 on tubular regeneration in AKI was regulated by transcription factor ZBTB7A

**Fig. 8:**
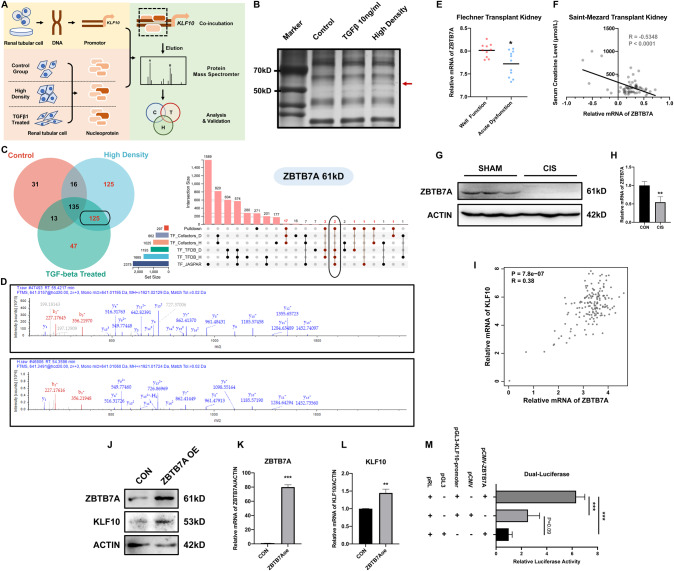
ZBTB7A promoted KLF10 expression by combining to the promotor regions of KLF10. **A** Flowchart detailing extraction and analyses of transcription factors combining KLF10 promoter in C57BL/6 J mice. **B** Silver staining SDS-PAGE gel of nucleoprotein precipitated with promoter of KLF10. **C** Venn plot of precipitated proteins in high-density, TGFβ1 treated group and control group. UpSetR plot of common transcription factors database and proteins only in high-density and TGFβ1 treated group not in control group (marked in red in Venn plot). **D** Protein mass spectrometer of ZBTB7A in high-density/TGFβ1 treated group and not detected in control group. **E** Scatter plot of relative expression of KLF10 in normal function group versus dysfunction group in Flechner database. **F** Negative correlation between ZBTB7A and SCr in Saint-Mezard database. **G**, **H** Representative immunoblot analysis and relative mRNA levels of ZBTB7A in cisplatin-induced AKI group and control group. ACTIN served as the standard. *n* = 6 per group. **I** Positive correlation between KLF10 and ZBTB7A in TCGA and GTEx normal kidney tissue database. **J**–**L** Representative immunoblot analysis and relative mRNA levels of ZBTB7A and KLF10 in ZBTB7A overexpressed group and control group. **M** Luciferase activity quantified in 293 T cells of interaction between ZBTB7A and KLF10 promoter. *p < 0.05, **p < 0.01, ***p < 0.001 vs control group at the same experimental conditions.

In view of the important role of KLF10 in the regulation of renal epithelial proliferation, we finally investigated the upstream of KLF10. Previous study and the results above showed that the expression of KLF10 in renal tubular cells could be induced by TGFβ1 treatment [27] and high-density culture condition. The nucleoprotein from control group, high-density cultured group and TGFβ1 treated group was extracted respectively and then co-incubated with the biotin-tagged promoter segment of KLF10. Through overnight incubation and purification, the nucleoprotein combining to the promoter of KLF10 was obtained (Fig. 8A). Silver staining SDS-PAGE gel of nucleoprotein precipitated with promoter of KLF10 uncovered a significant enhanced band around 60 kD in both high-density cultured group and TGFβ1 treated group versus the control (Fig. 8B). After identification by protein mass spectrometry assay, the protein in both high-density cultured group and TGFβ1 treated group and exclusive of protein from control group was obtained (N1 = 125). All of the pulldown protein in classical transcription factor databases (TFDB [37] and JASPAR, https://jaspar.genereg.net, [38]) exclusive of protein from control group was then obtained (N2 = 2). The intersection between N1 and N2 was ZBTB7A (Fig. 8C), which could be detected in both high-density cultured group and TGFβ1 treated group instead of control group (Fig. 8D). Thus, ZBTB7A might be the transcription factor combining to the promoter of KLF10 especially when the proliferation of renal tubular cell slowed down.

Then, we explored public transcriptomic data [31, 39] and found that ZBTB7A decreased significantly in acute dysfunctional kidney versus well-functioning kidney and negatively correlated to SCr (Fig. 8E, F). Consistent with the expression pattern of KLF10, the declining of ZBTB7A was further validated in cisplatin-, CLP- and IR-induced AKI mouse models (Fig. 8G/H and Supplementary Figs. 2D/E, 3D/E). Therefore, ZBTB7A also took part in the progression of AKI as KLF10 did.

In order to validate the transcription factor role of ZBTB7A to KLF10, transcriptomic data of kidney para-carcinoma tissue in TCGA and normal kidney tissue in GTEx from GEPIA [33] showed significant positive correlation between KLF10 and ZBTB7A (Fig. 8I). Moreover, overexpression of ZBTB7A in 293 T induced the expression of KLF10 (Fig. 8J–L). Furthermore, luciferase activity of the 293 T cells was significantly activated when co-transfecting pGL3-KLF10-promoter and pCMV-ZBTB7A, confirming that ZBTB7A promoted the expression of KLF10 by combing to its promoter (Fig. 8M). Taken together, proliferative inhibitive effect of KLF10 in AKI was positively regulated by transcription factor ZBTB7A.

## Discussion

Cell proliferation is the key process for tissue regeneration after injury. Cytokines and mechanisms that promote renal epithelial cell proliferation were well studied [14, 19–25], but factors that inhibit or cease the proliferate process were rarely defined. In this study, we provided novel evidence showing that renal epithelial cell down-regulated ZBTB7A in response to injury, and then decreased KLF10 expression which further inhibited PTEN/AKT signaling for initiating cell proliferation. We first unveiled the downregulation of KLF10 in human renal transcriptomic database of acute kidney dysfunction, 3 classical AKI mouse models and 3D AKI tubular model, which suggested evidently that KLF10 was closely related to renal function and tubular proliferation. Consistently, as injury alleviated in AKI model in vivo and in vitro, KLF10 rose again. KLF10 also elevated obviously in the group with contact inhibition and the late stage of tubular formation. Overexpression or knockdown of KLF10 directly influenced the capacity of renal tubular cells to proliferate, form lumen and repair, which further clarified the causal connection between KLF10 and tubular regeneration. Moreover, ZBTB7A-KLF10-PTEN axis was found to be the specific mechanism of regenerative regulation of KLF10.

The transplant kidney data were first adopted for analysis, since the resource of human kidneys with common AKI has been always limited due to such kidneys being seldomly biopsied. Instead, kidney transplants become the valuable resources, for kidney transplants often experience AKI and are extensively followed up with detailed clinical data and kidney punctures, even in the well-functioning stages [39, 40]. Considering that we aimed to excavate the renal injury and renal function related changes alone rather than changes owing to transplant process or drug usage, the kidneys from healthy living donor not conquered transplant/ drug attack were excluded. Well-functioning transplants were used as the control instead. Therefore, the downregulation of KLF10 and its clinical meaning for renal function were demonstrated convincingly from realistic human data to experimental multiple mouse models in vivo, and finally to cellular model in vitro (Figs. 1–3). Moreover, transcriptomic data from tubulointerstitium of other kidney diseases showing decreasing KLF10 revealed the significant role of KLF10 in renal tubules.

Renal repair process is always the front burner topic in the area of kidney injury, especially in AKI. Tubular injury and regeneration, the most common issue in AKI, thereby becomes the vital core in the pathophysiological process of AKI [10, 11]. However, only AKI model in vivo cannot distinguish the exact changes in renal tubules from other tissue parts and common cellular culture is far from the condition in vivo for loss of epithelial polarity. Thus, we constructed 3D tubular injury model in vitro to mimic the conditions in AKI progress in vivo, which was much more persuasive. As the manifestation in vivo, cell shedding, apoptosis and necrosis was also observed in 3D tubular model (Fig. 2). Moreover, we separated the survived cell from injury dead cells to specify the changes of KLF10 (Fig. 4). Furthermore, Fucci cell cycle visualization cellular system was also applied into our model, which could monitor the proliferation activity continuously in a real-time living way [32]. And when tubular proliferation slowed down in high-density culture condition and the late stage of tubular formation, KLF10 elevated significantly (Fig. 5).

For the experiments above only verified the simultaneity between KLF10 and tubular regeneration in AKI, overexpression and knockdown of KLF10 were carried out to validate their causal relationship. The ability to proliferate, repair and form lumen were all inhibited remarkably (Fig. 6), whereby KLF10 might also get involved in the renal development and self-renewal process.

To specify the exact mechanism, PTEN/AKT signaling was focused on for its roles of tumor suppressor and downstream of KLF10 in previous tumor study [28, 34, 35] and the same change of expression as KLF10 in AKI. At the same time, elevation of PTEN was examined in physical inhibition model and the late stage of tubular formation. Moreover, inhibiting PTEN could reverse the negative effect of KLF10 on tubular regeneration in cisplatin-induced AKI, which confirmed the downstream role of PTEN/AKT signaling (Fig. 7). Interestingly, phosphorylation of AKT increased at the late stage of tubular formation, which might be due to other effects of pAKT in lumen formation and needs further study. In the meanwhile, the upstream transcription factor ZBTB7A was found by protein mass spectrometer assay of nucleoprotein precipitated with the promoter of KLF10. Previous study has reported that ZBTB7A suppressed the proliferation of several epithelial tumor [41, 42]. Thus, other than the same expression pattern as KLF10 in vivo, the transcription factor role of ZBTB7A was validated by dual-luciferase reporter assay, which integrated the up- and down-stream regulative mechanism of KLF10.

There were also several limitations in our study. The human samples in this study were from public database and limited to the transcriptomic level, lacking further validation of protein levels due to unavailability to samples of healthy and AKI human kidney. Meanwhile, mouse models in vivo were not further validated by employing KLF10 knockout mice due to time and financial constraints. The main object of this study was cisplatin-induced AKI and in the in vivo models, we also extended our idea in IRI- and CLP-induced AKI, suggesting the potential universal significance of KLF10 in AKI with various etiology. However, only cisplatin was used to induce 3D tubular injury in our in vitro study owing to the limitation of equipment for hypoxia culture and huge differences of the LPS treated 3D tubular model in vitro from sepsis model in vivo, which would be explored and constructed in the future when available.

## Conclusions

Collectively, our study proposed a novel mechanism wherein downregulation of KLF10 contributed to the proliferation of survived renal tubular cells in cisplatin-induced acute kidney injury via ZBTB7A-KLF10-PTEN axis, which might be a promising diagnostic and therapeutic target of AKI.

## Methods

### Data collection, weighted gene co-expression network analysis, functional enrichment analysis, and code availability

GSE1563 dataset was obtained from the GEO database (https://www.ncbi.nlm.nih.gov/geo/) [31], WGCNA R package (version: 1.70-3) was performed to construct a scale-free network. The soft-thresholding power was set as 7 with a correlation coefficient of 0.8. Cluster analysis divided highly correlated genes into gene modules after proper infiltration. After topological overlap matrix (TOM) was obtained, the data was transferred to the network into the edge and node list files Cytoscape could read. Subsequently, the network diagrams were plotted by Cytoscape 3.9.0. Functional enrichment analysis was carried out by Metascape (https://metascape.org) [43]. Renal tubulointerstitial RNA-seq data of other kidney diseases were obtained from Nephroseq database (www.nephroseq.org, version November 2022, University of Michigan, Ann Arbor, MI). The tutorial and source code of WGCNA could be found on the website (https://horvath.genetics.ucla.edu/html/CoexpressionNetwork/Rpackages/WGCNA/Tutorials/).

### Animal models

Male C57BL/6 J mice (7–9 weeks of age, weighing 20–25 g) were obtained from Shanghai Jihui Laboratory Animal Care Co. LTD, Shanghai, China. All the protocols were approved by the Animal Care and Use Committee of Zhongshan Hospital and were performed in accordance with the National Institutes of Health Guide for the Care and Use of Laboratory Animals. All the experiments were replicated at least twice and mice were randomized to each group.

To establish IR-induced AKI model, bilateral renal pedicles were clamped for 30 min for mice in the IR group with mice body temperature retained at 35–36 °C during all surgical procedures. The same operation except clamping of renal pedicles for mice in the sham group. Kidneys were collected for analysis at different time points after reperfusion.

To establish the cisplatin-induced AKI model, mice in the cisplatin group were intraperitoneally injected with a single dose of cisplatin (Sigma–Aldrich) at 20 mg/kg and mice of the control group were received saline alone. Kidneys were collected for analysis at different time points after treatment.

To establish the sepsis-induced AKI model, the cecum was exteriorized and ligated distal of the ileocecal valve. Then, the cecum was perforated three times using a 20-gauge needle and squeezed to extrude fecal contents that were spread around the cecum using a cotton swab, with mice body temperature retained at 35–36 °C during all surgical procedures. In sham animals, the cecum was exteriorized without ligation and puncture. Kidneys were collected for analysis at different time points after surgery.

### Cell culture and treatment

Madine-Darby Canine Kidney (MDCK) cells were purchased from the American Type Culture Collection. Cells were cultured in Minimum Essential Medium Eagle (Sigma-Aldrich) supplemented with 5% FBS (Gibco) under the condition of 37 °C, 5% CO_2_ and saturation humidity. The plasmids harboring KLF10/NC siRNA manufactured by Shanghai Genechem Co., Ltd and lentivirus vectors harboring CDS of KLF10 and GFP/puromycin-resistance and GFP/control manufactured by HanBio Co, Ltd, Shanghai, China were transfected into cells according to the manufacturer’s guidelines.

MDCK cells were also used for the 3D culture. The Matrigel matrix (Corning, USA) and the cells were mixed well and spread on a Matrigel matrix-coated culture plate. After culturing for 5–6 days, 3D renal tubules were formed.

MDCK-Fucci cells were kindly provided by professor Cai Liang in school of life sciences at Fudan University, which would show red fluorescence during G2 to M phase.

Cisplatin was dissolved to 1 mg/mL in saline and diluted with MEM to different final concentrations before usage. Cells were collected at different time points after cisplatin treatment. Recombinant TGFβ1 (10804-HNAC, SinoBiological) was dissolved to 2 μg/mL in in sterile 4 mM HCl containing 1 mg/mL bovine serum albumin and diluted with MEM to different final concentrations before usage. Cells were collected 24 h after TGFβ1 treatment. PTEN specific inhibitor bpV (HOpic) (S8651, Selleck) was dissolved to 1 mM in ddH_2_O and diluted with MEM to different final concentrations before usage. Cells were collected 72 h after bpV treatment.

### Western blot analyses

Proteins from cultured cells or mice kidneys were extracted with TRI Reagent (Sigma–Aldrich) according to the manufacturer’s guidelines. Samples were separated by SDS-PAGE and transferred onto PVDF membranes (IPVH00010, Millipore). After blocking the membranes with 5% milk, we used primary antibodies to incubate the membranes overnight at 4 °C against the following proteins: KLF10 (1:1000, ab184182, Abcam), CCNB1 (1:1000, GTX100911, GeneTex), CCND1 (1:1000, GTX108624, GeneTex), PCNA (1:1000, 101239-T46, SinoBiological), KIM1 (1:1000, AF1817, R&D), Anti-FLAG (1:1000, 8146, CST), ACTIN (1:2000, GTX11003, GeneTex), ZBTB7A (1:1000, ab175918, Abcam), PTEN (1:1000, AB170941, Abcam), pAKT (1:1000, 4060 S, CST), AKT (1:1000, 4691 S, CST). The membranes were washed by TBST before incubated with secondary antibodies (1:2000, Jackson ImmunoResearch Inc). The bands of the target proteins were visualized by the LAS-3000 detection system and were quantitively analyzed by Fiji based on ACTIN and control groups respectively.

### Real-time RT-PCR

Total RNA from MDCK and kidney tissues were extracted using TRI Reagent (Sigma–Aldrich). The reverse transcription and real-time RT-PCR were carried out using PrimeScript RT Master Mix and SYBR Premix ExTaq^TM^ (TaKaRa) on QuantStudio 5 (Thermo Fisher Scientific). Gene expression was measured relative to ACTIN or GAPDH and 2^−ΔΔCt^ method was used to calculate the fold change differences of the experimental groups compared to the control group. The following primers (Sangon Biotech) 5’ to 3’ were used:

**Table:** 

*Canis lupus familiaris*		
KLF10	F	CTCCCGGGTACACCTGATTTT
	R	GCAATGTGAGGCTTGGCAGTATC
ACTIN	F	TGCGGCATCCATGAAACTAC
	R	ACAGCACTGTGTTGGCATAG
*Mus musculus*		
KLF10	F	ATGCTCAACTTCGGCGCTT
	R	CGCTTCCACCGCTTCAAAG
HAVCR1	F	AGGCGCTGTGGATTCTTATG
	R	AAGCAGAAGATGGGCATTGC
LCN2	F	TGGCCCTGAGTGTCATGTG
	R	CTCTTGTAGCTCATAGATGGTGC
IGFBP7	F	TAACCTGCGAATCCATGAGC
	R	AGAGAAGTGTGTCAGGCAAGAG
PCNA	F	TTTGAGGCACGCCTGATCC
	R	GGAGACGTGAGACGAGTCCAT
FOXM1	F	GGACATCTACACTTGGATTGAGG
	R	TGTCATGGAGAGAAAGGTTGTG
MKi67	F	ATCATTGACCGCTCCTTTAGGT
	R	GCTCGCCTTGATGGTTCCT
GAPDH	F	AGGTCGGTGTGAACGGATTTG
	R	GGGGTCGTTGATGGCAACA
ACTIN	F	AGCCATGTACGTAGCCATCC
	R	GCTGTGGTGGTGAAGCTGTA
*Homo Sapiens*		
ZBTB7A	F	GCTTGGGCCGGTTGAATGTA
	R	GGCTGTGAAGTTACCGTCGG
KLF10	F	CTTCCGGGAACACCTGATTTT
	R	GCAATGTGAGGTTTGGCAGTATC
ACTIN	F	TCACCCACACTGTGCCCATCTACGA
	R	CAGCGGAACCGCTCATTGCCAATGG

### Immunofluorescence and hematoxylin-eosin (H&E) staining

After treatment, renal tissues were fixed with 4% paraformaldehyde, embedded in paraffin wax and sliced into 3-μm-thick sections for hematoxylin–eosin (H&E) staining. The slides were incubated in the mixed primary antibodies overnight at 4 °C: KLF10 (1:100, 11881-1-AP, Proteintech) and Ki67 (1:50, GB111141, Servicebio). The slices were then incubated with Alexa Fluor® 488-conjugated goat anti-rabbit IgG (1:200, ab150077, Abcam). Nuclei were stained with 4,6-diamidino-2-phenylindole (DAPI).

After treatment, MDCK cells which crawled on the slide were fixed in 4% paraformaldehyde for 15 min. Cells were permeabilized by 0.5% Triton X-100 in PBS for 10 min. After blocking with 5% BSA in PBS, sections or cells were incubated with primary antibody against KLF10 (1:100, 11881-1-AP, Proteintech) followed by Alexa Fluorophore 488-conjugated secondary antibody (1:200, abs20020, Absin Bioscience). Nuclei were stained with 4,6-diamidino-2-phenylindole (DAPI). The results were visualized by Olympus microscope (Tokyo, Japan) and the light microscopy (Leica DM 6000B; Leica Microsystems, Wetzeler, Germany).

### Serum creatine ELISA

Serum creatinine levels of mice were determined using a QuantiChrom^TM^ Creatinine Assay Kit (BioAssay Systems, Hayward, CA, USA) following the manufacturer’s guidelines.

### Cell proliferation assay

2000 MDCK cells overexpressed KLF10 or control were seeded into a 96-well plate per well. CCK-8 reagent (Dojindo) was added after treatment and incubated for 90 min at 37 °C. Cell viability was assessed by absorbance at 450 nm using the spectrophotometer (Thermo Fisher Scientific) 0, 24, 48 and 72 h after adherence.

### Wound healing assay

4 × 10^5^ MDCK cells overexpressed KLF10 or control were seeded into 6-well plate per well and cultured until 100% confluence. Then confluent cultures were scratched using a pipette tip. After scratching, the dishes were gently washed twice with medium to remove the detached cells. Scratched cultures were photographed under a microscope at 0, 12 and 24 h. Capacity to repair of cells was established by measuring the width of the scratched area at each time point in the scratched area.

### DNA pulldown, nucleoprotein extraction and mass spectrometry

Biotin labeled KLF10 promoter DNA was prepared by PCR amplifying using forward primer: 5’-biotin/TTCTCGAGTCACAAGTCAAGACCGCTCCCT-3’ and reverse primer: 5’-biotin/ TTAAGCTTTTGAGCTCGGTGTAGCTGAAGTTTAAA-3’. The resulting 1100 bp DNA fragment were further gel extracted and purified by DNA gel extraction kit (AxyPrepTM, Ap-GX-25). MDCK nuclear extracts were prepared using NE_PER Nuclear and Cytoplasmic Extraction kit (Thermo Fisher, 78833), and quantified by BCA. Briefly, following nuclear extraction, 1 mg of lysate was incubated with 3 pmol of biotinylated DNA along with 120 μL of streptavidin magnetic beads (Thermo Fisher, 88816). The final volume was adjusted to 500 μL using NER buffer. The mixture was incubated in rocker at 4 centigrade degrees for 24 h. The samples were placed on a magnetic stand and washed with ice cold PBS three times followed by one wash with NER buffer. The beads were resuspended in 16 μL PBS and 4 μL 5x SDS sample buffer, boiled at 70 °C for 10 min and the proteins were separated by SDS-PAGE. The gel was visulized by silver staining and identified by mass spectrometry (MS) (H. Wayen Biotechnology, Shanghai). The silver-stained gel was treated with decolorization, reductive alkylation, enzymolysis and desalination in order before MS examination. The samples were then isolation by EASY-nLC 1000 (Thermo Scientific, USA), examined by Orbitrap Fusion Lumos (Thermo Scientific, USA) and analyzed by Proteome Discoverer 2.4 software (Sequent HT), (Thermo Scientific, USA) for MS.

### Dual-luciferase activity assay

KLF10 promoter were first cloned into the dual-luciferase reporter plasmid vector and then was co-transfected with pCMV-ZBTB7A or negative control (Shanghai Genechem Co., Ltd) into 293 T cells. Dual-luciferase activity was measured using the Dual-Glo Luciferase Assay System (E2920, Promega).

### Statistical analysis

Data were expressed as means ± standard error of the mean. Two-tailed unpaired *t*-tests, Two-Way ANOVA, correlation and linear regression were performed using Microsoft Excel for Mac and GraphPad Prism 9.0 software for Mac. ns *p* ≥ 0.05, *p < 0.05, **p < 0.01, ***p < 0.001, ****p < 0.0001 vs control group at the same experimental conditions.

## Supplementary information

Supplementary Figures

Supplementary Mass Spectrometry Data

Original Data File

## Data Availability

All data can be available which are specified in “Methods”.
