# Supplementary material for: Downregulation of KLF10 contributes to the regeneration of survived renal tubular cells in cisplatin-induced acute kidney injury via ZBTB7A-KLF10-PTEN axis
Source: Cell Death Discov. 2023 Mar 6;9:82. doi: 10.1038/s41420-023-01381-6 (PMC9988960; doi:10.1038/s41420-023-01381-6)
Supplement: Supplementary file 1 — Supplementary Figures [file 41420_2023_1381_MOESM1_ESM.docx]

**Supplementary Material and Methods**

**
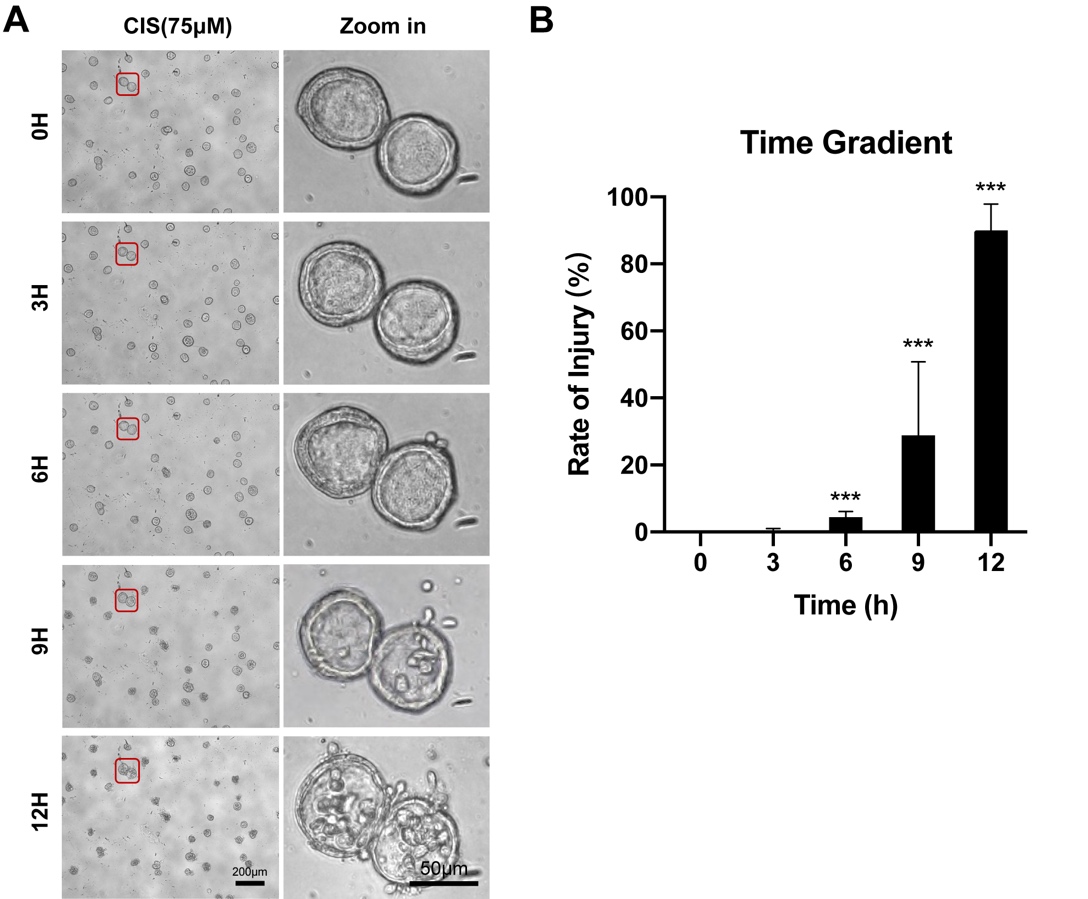
**

**Supplementary figure 1.** The morphology (A) and injury rate (B) of 3D renal tubular model treated with cisplatin for different duration. ***p<0.001 vs control group at the same experimental conditions.

**
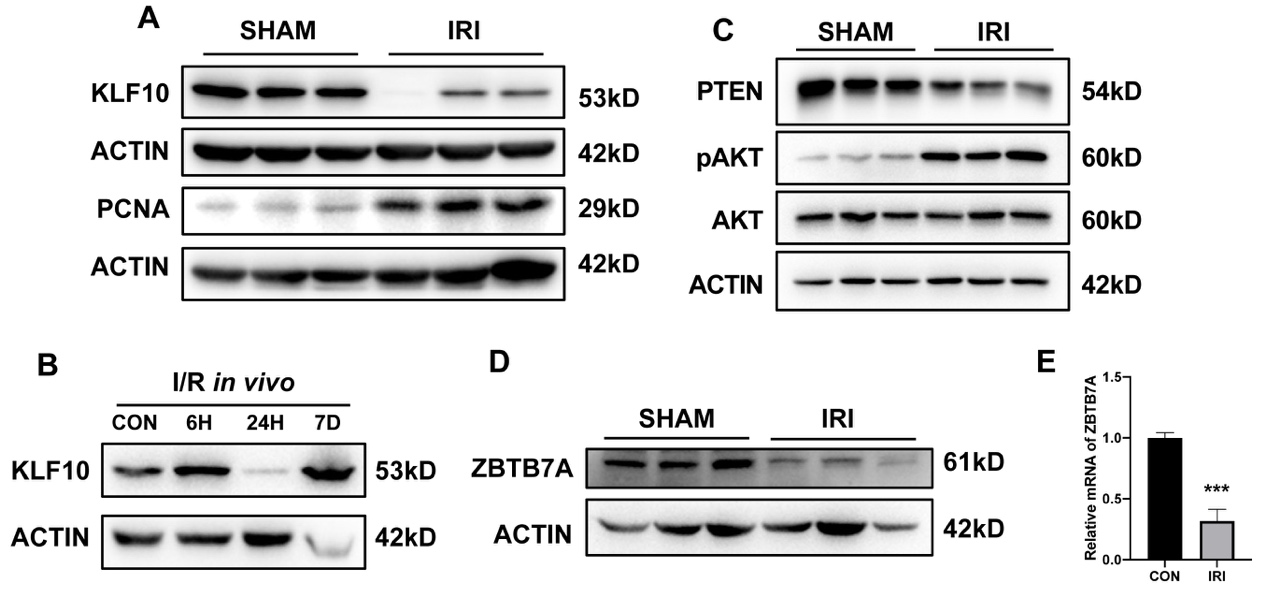
**

**Supplementary figure 2.** (A) Representative immunoblot analysis of KLF10 and PCNA in kidney tissues from IR-induced AKI mouse model. ACTIN served as the standard. (B) Representative immunoblot analysis of KLF10 in mice sham, 6hrs, 24hrs and 7d after bilateral 30mins IR treatment. ACTIN served as the standard. (C) Representative immunoblot analysis of PTEN, pAKT and AKT in IR-induced AKI group and sham group. ACTIN served as the standard. (D, E) Representative immunoblot analysis and relative mRNA levels of ZBTB7A in IR-induced AKI group and sham group. ACTIN served as the standard. n = 6 per group. ***p<0.001 vs control group at the same experimental conditions.

**
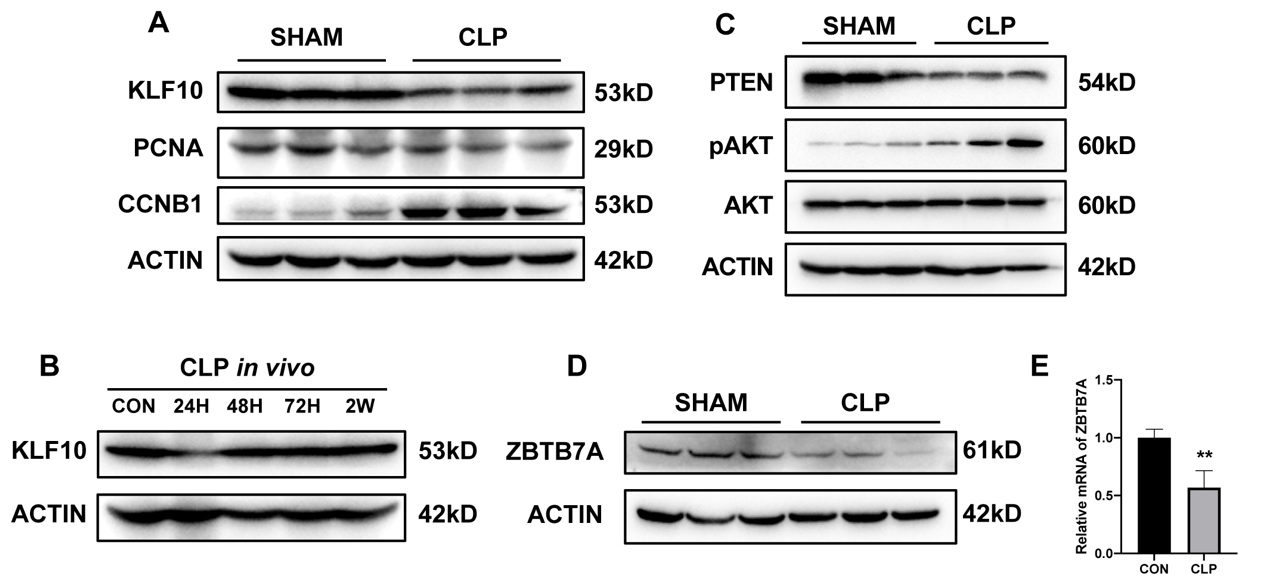
**

**Supplementary figure 3.** (A) Representative immunoblot analysis of KLF10, PCNA and CCNB1 in kidney tissues from CLP-induced AKI mouse model. ACTIN served as the standard. (B) Representative immunoblot analysis of KLF10 in mice sham, 24hrs, 48hrs, 72hrs and 2wks after CLP treatment. ACTIN served as the standard. (C) Representative immunoblot analysis of PTEN, pAKT, AKT in CLP-induced AKI group and sham group. ACTIN served as the standard. (D, E) Representative immunoblot analysis and relative mRNA levels of ZBTB7A in CLP-induced AKI group and sham group. ACTIN served as the standard. n = 6 per group. **p<0.01 vs control group at the same experimental conditions.

**
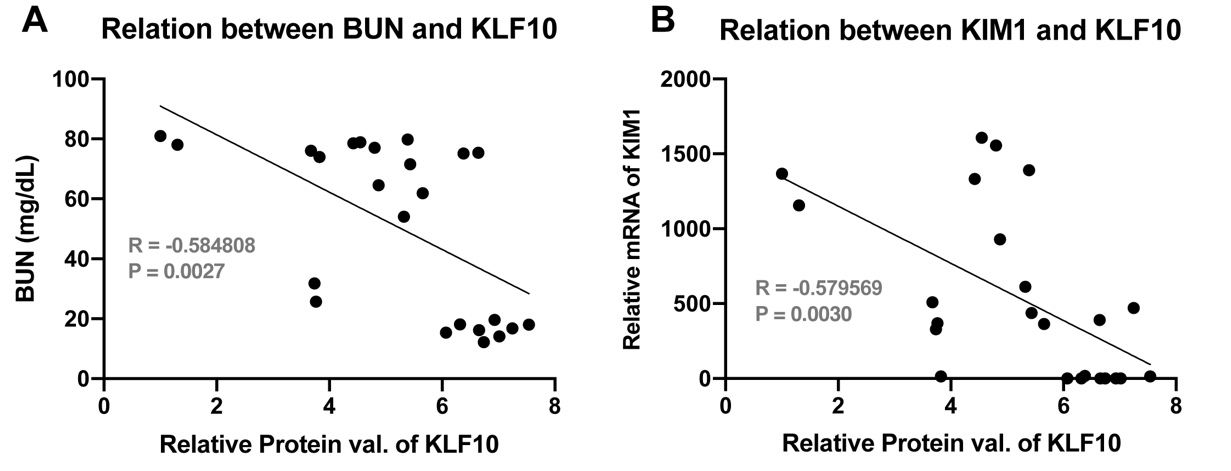
**

**Supplementary figure 4.** Regression analysis was undertaken to determine correlation between relative protein level of KLF10 in mice kidney tissues and BUN (A) /KIM1 (B). Relative protein level of KLF10 was examined and normalized by Fiji.
