# Supplementary material for: Downregulation of KLF10 contributes to the regeneration of survived renal tubular cells in cisplatin-induced acute kidney injury via ZBTB7A-KLF10-PTEN axis
Source: Cell Death Discov. 2023 Mar 6;9:82. doi: 10.1038/s41420-023-01381-6 (PMC9988960; doi:10.1038/s41420-023-01381-6)

Figure 2B

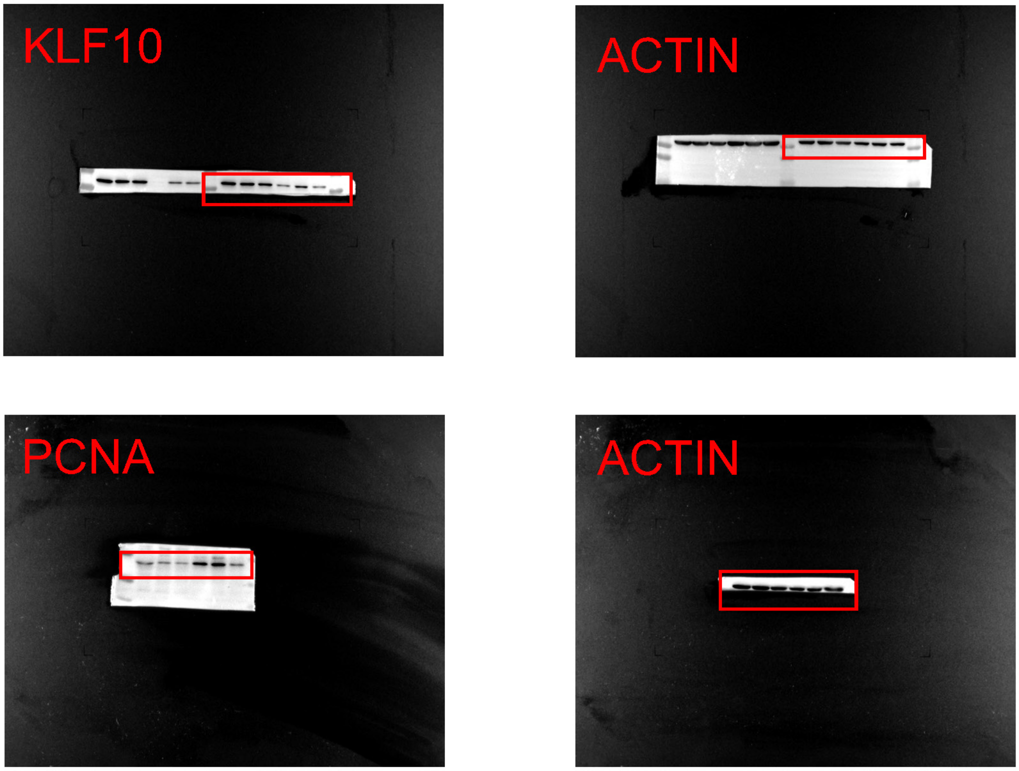


Figure 2E

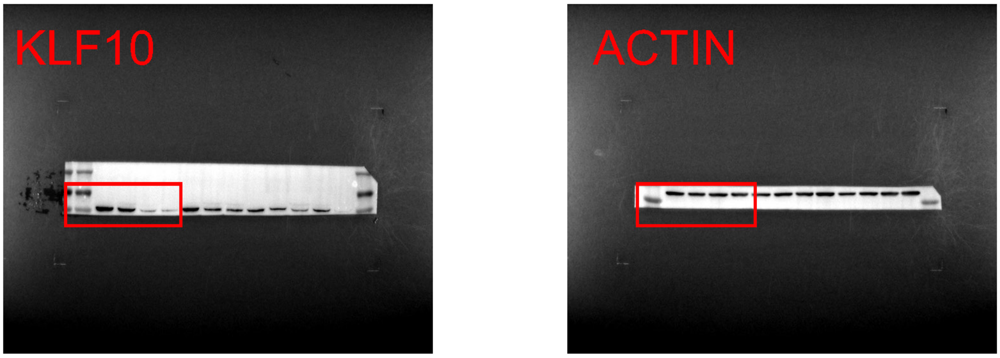

Figure 3E

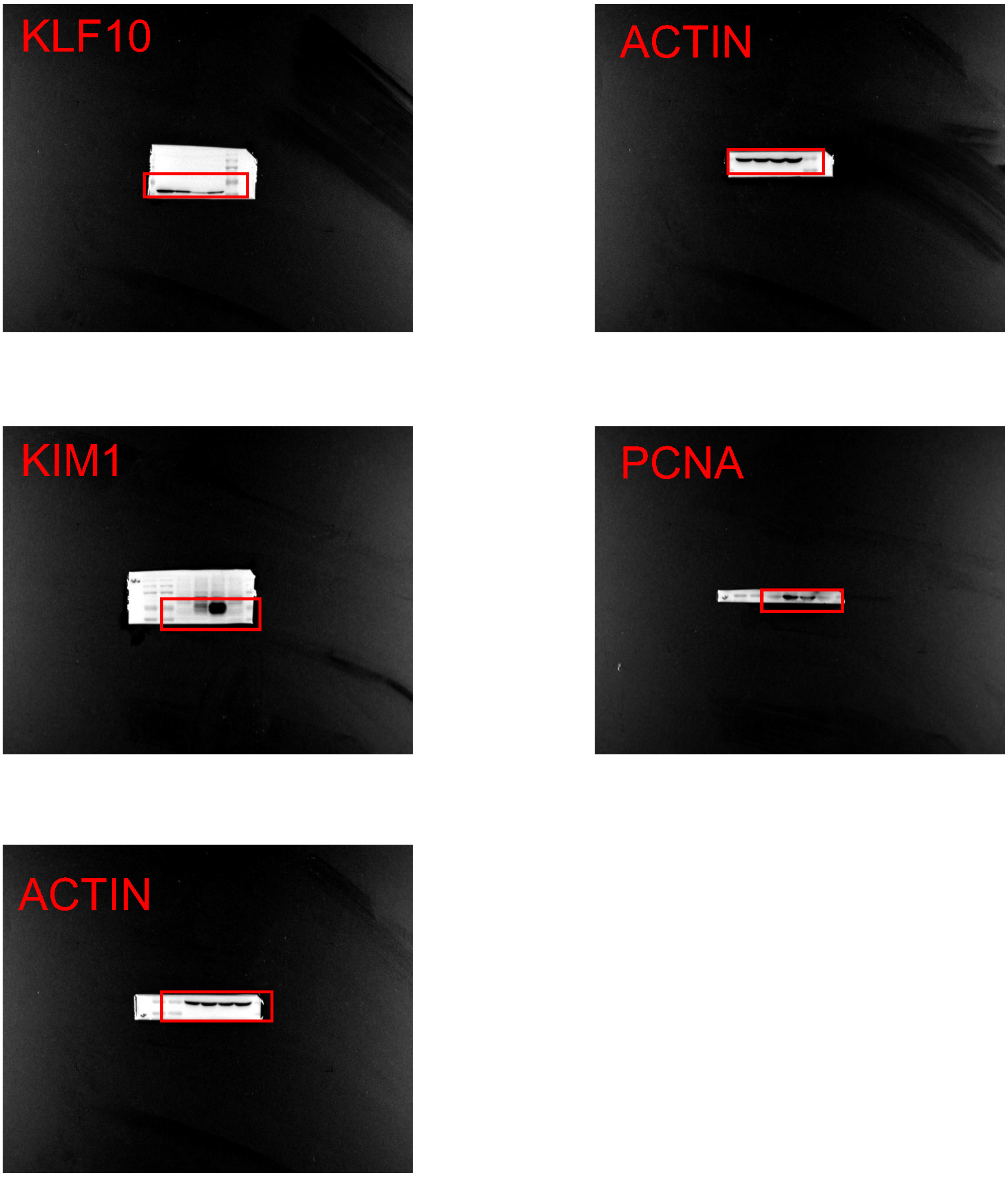

Figure 4B

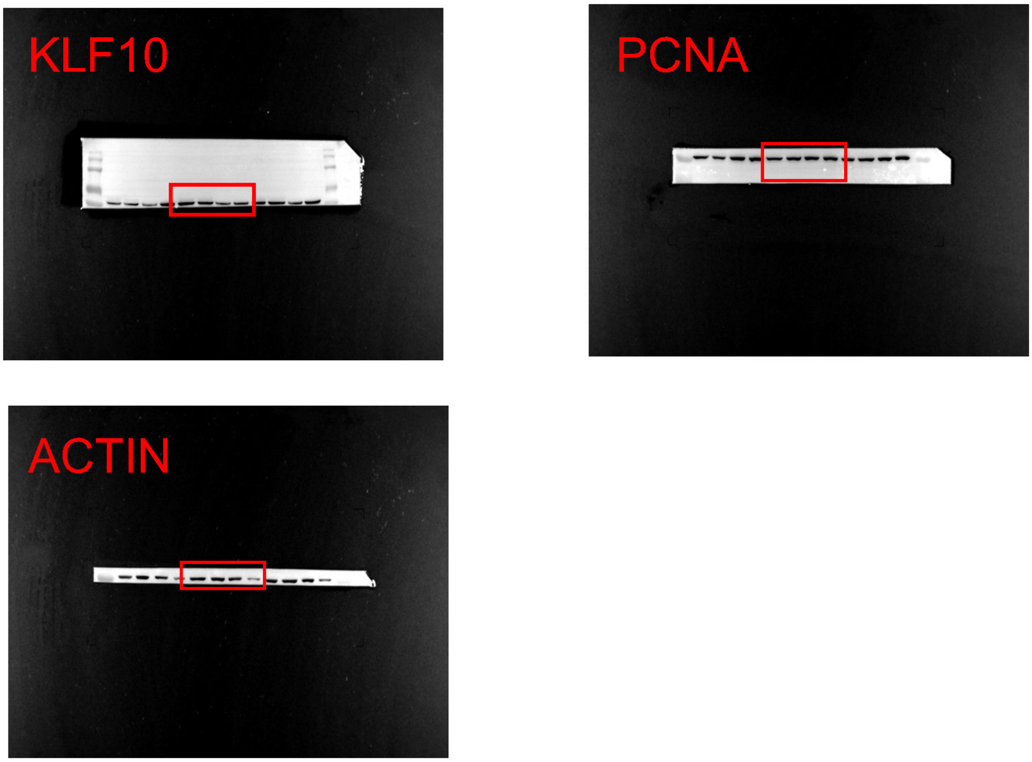


Figure 4E

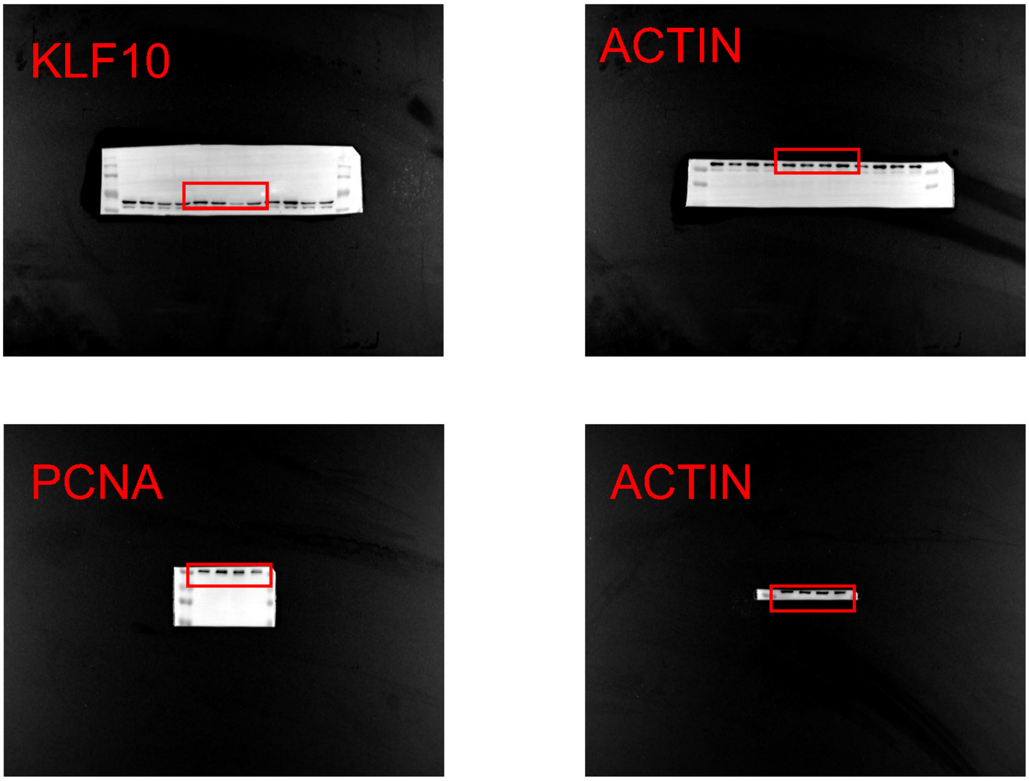

Figure 5C

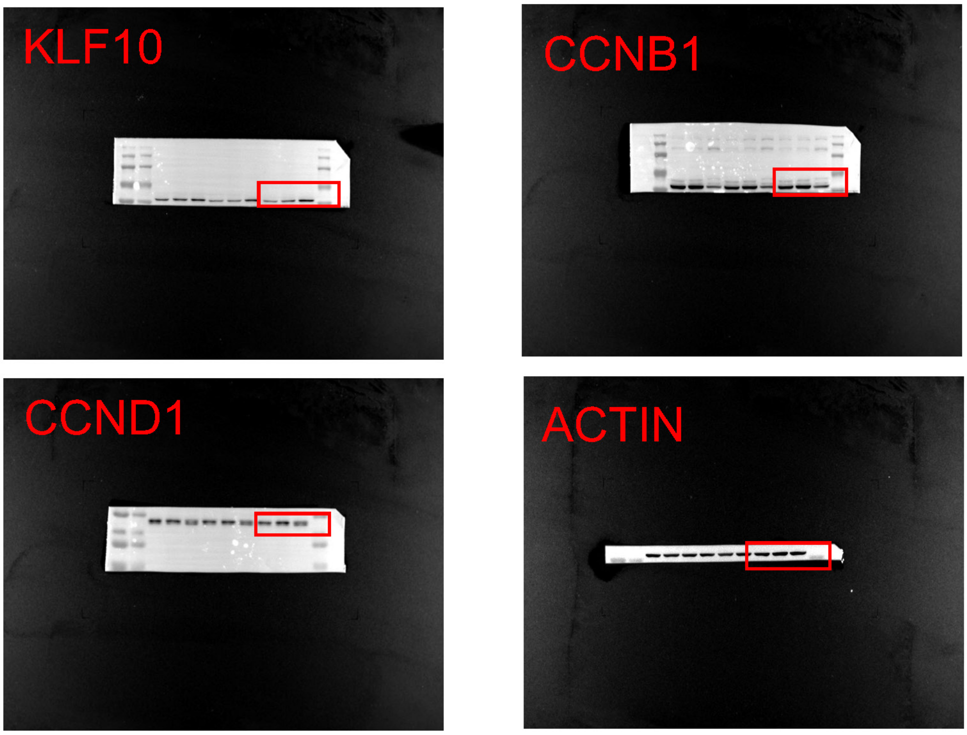


Figure 5E

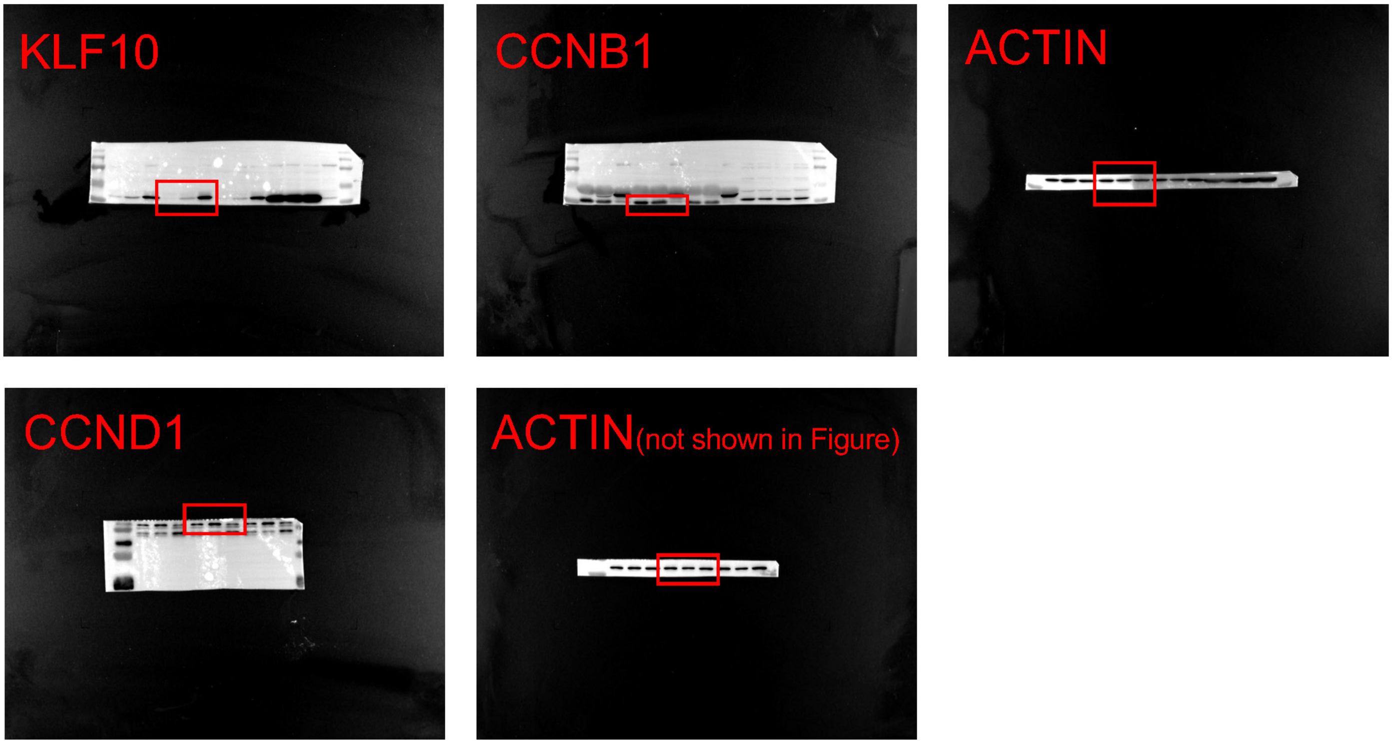

Figure 6A

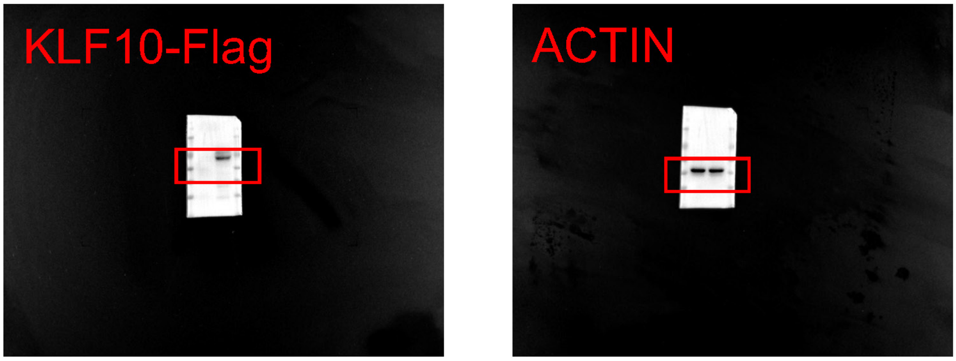


Figure 6C

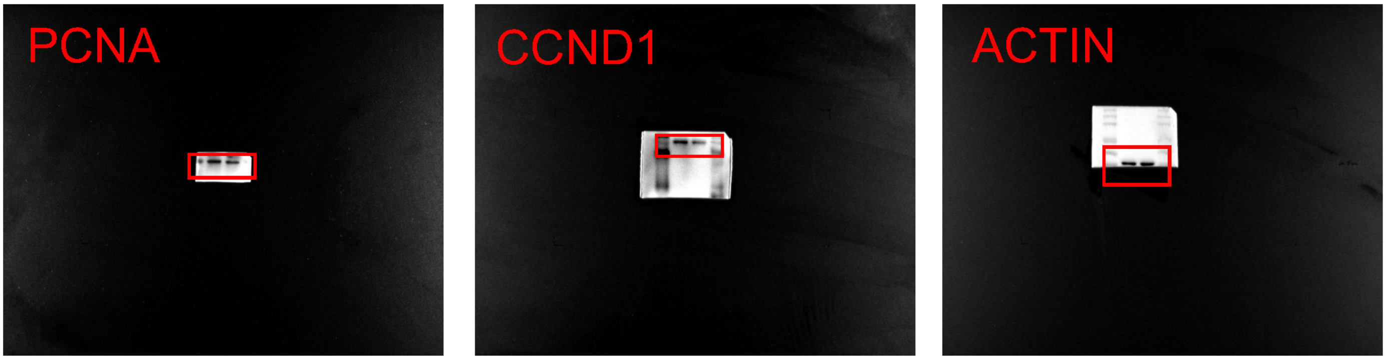


Figure 6K

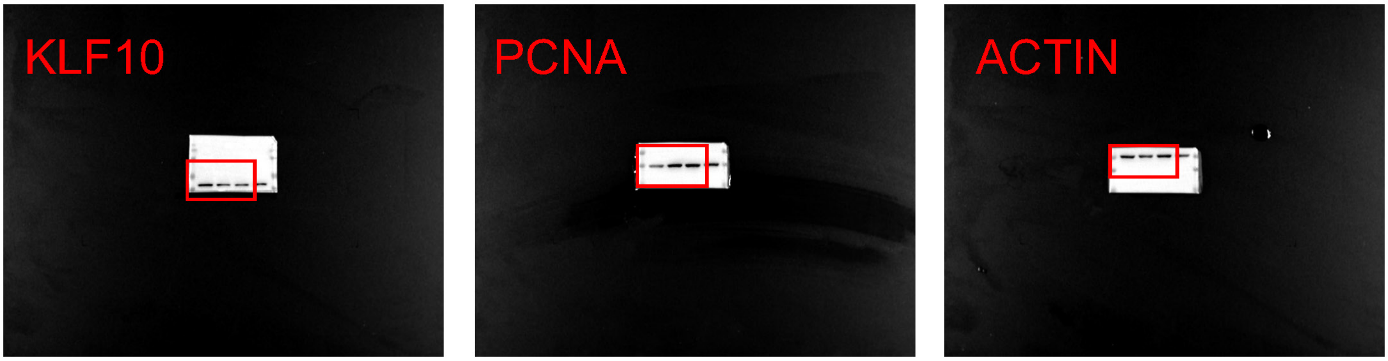

Figure 7A

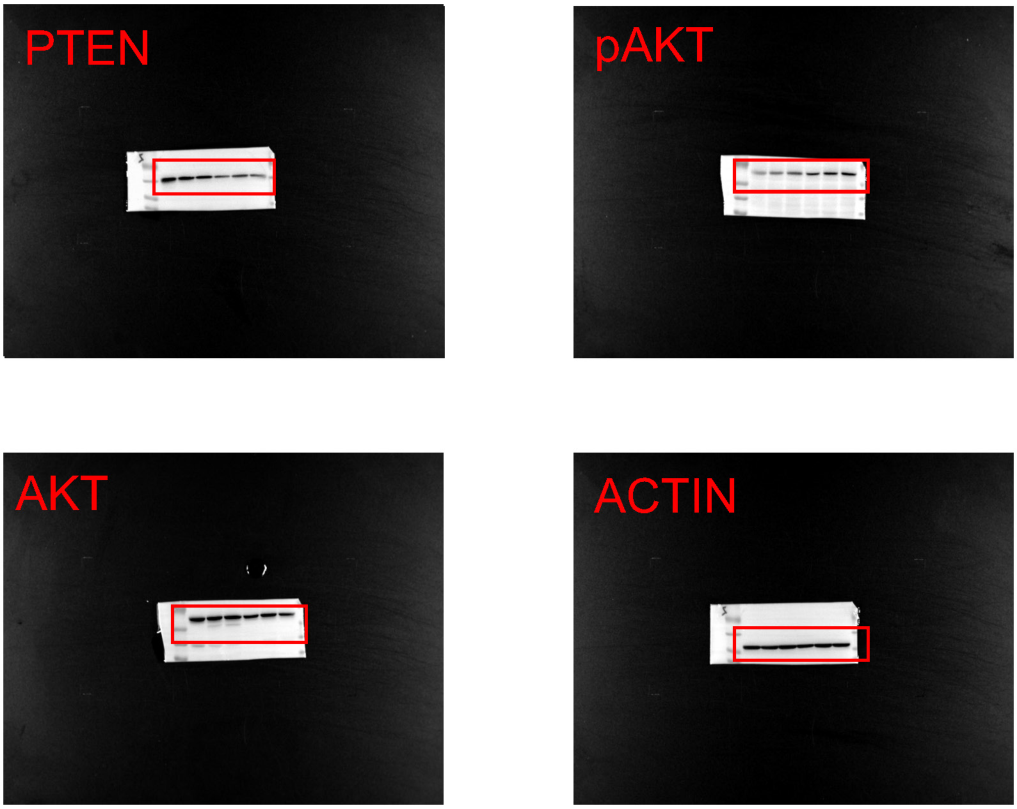


Figure 7B

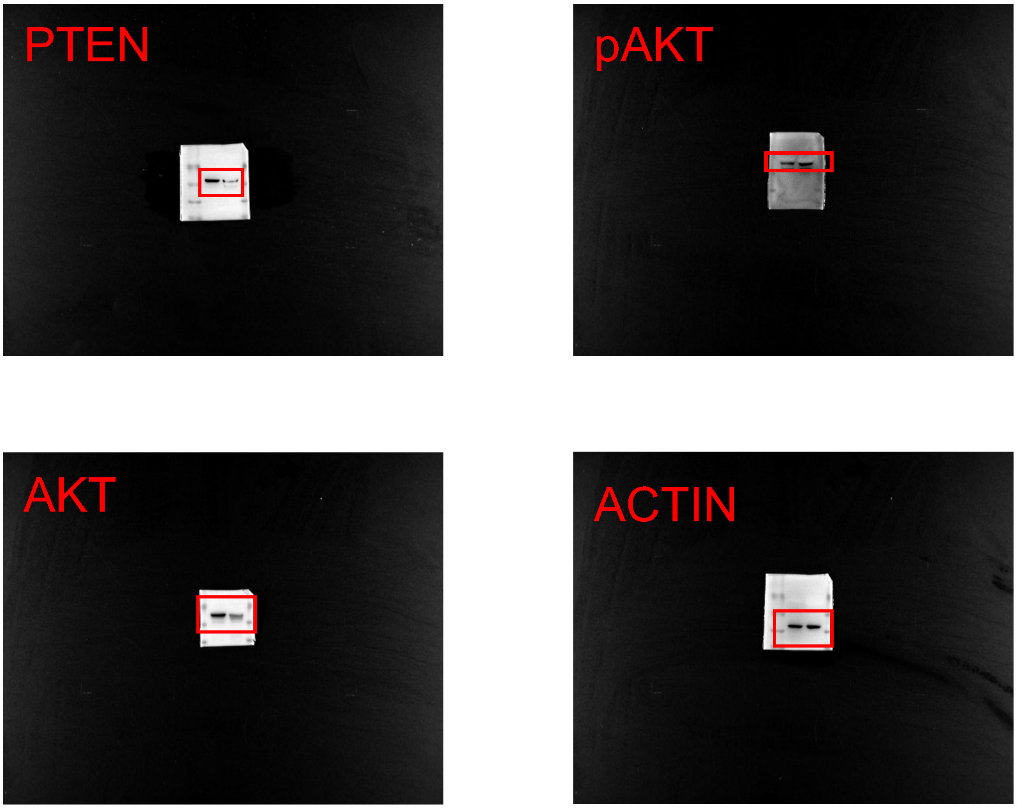

Figure 7C

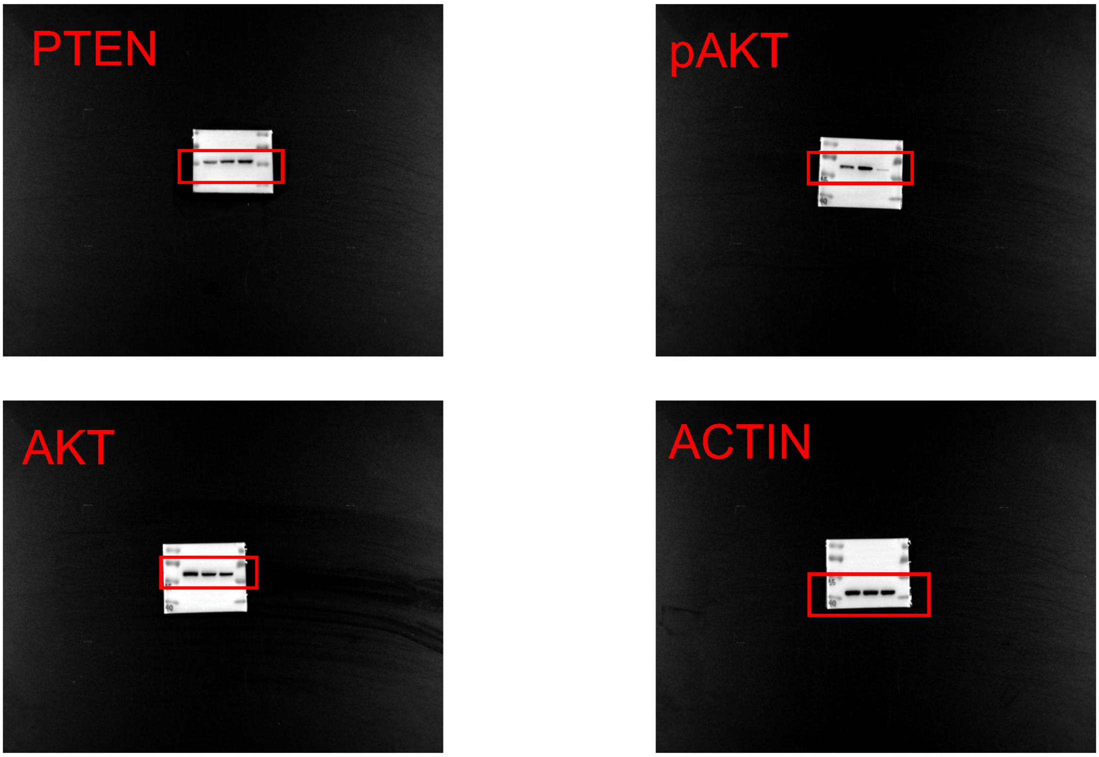


Figure 7D

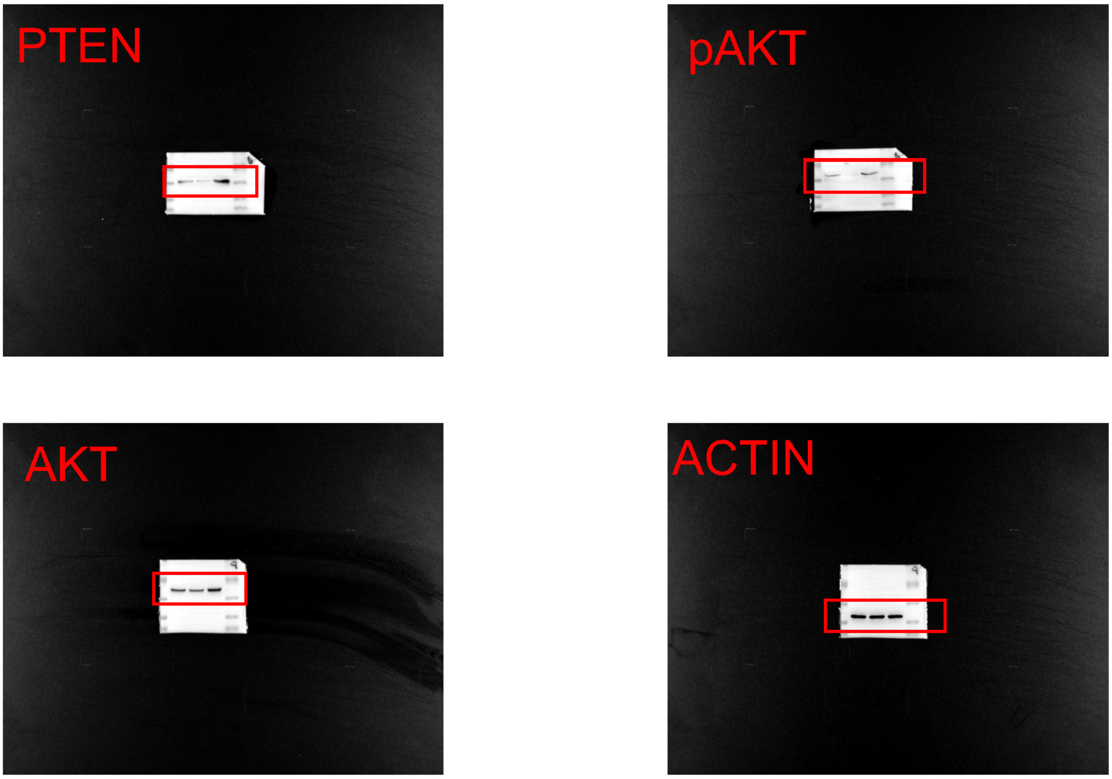

Figure 7E

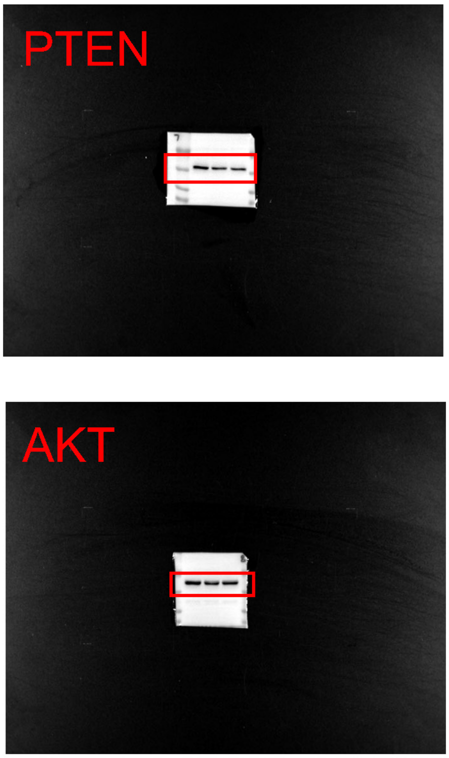


Figure 7F

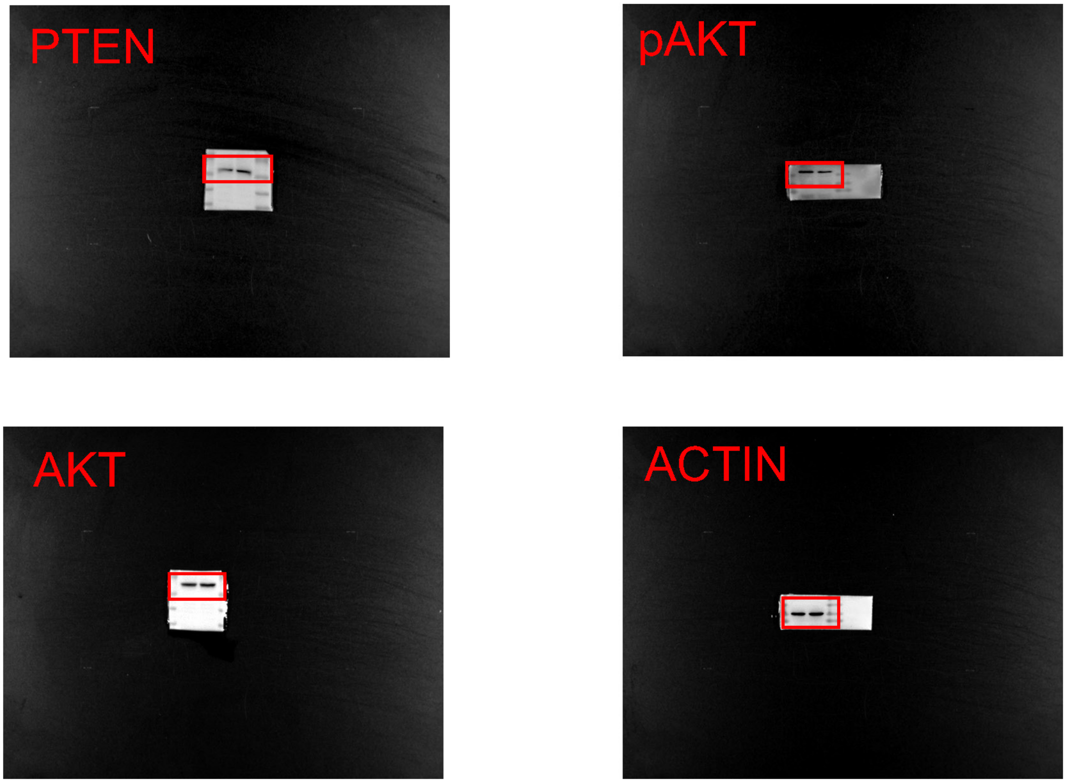

Figure 7G

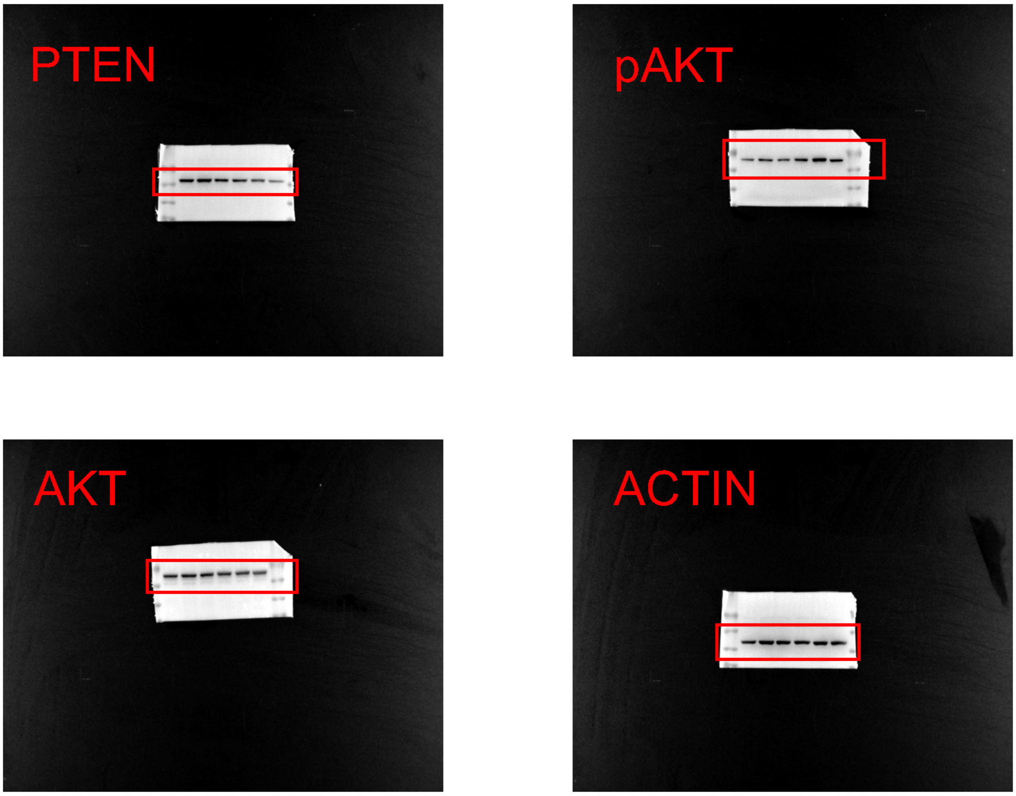


Figure 8G

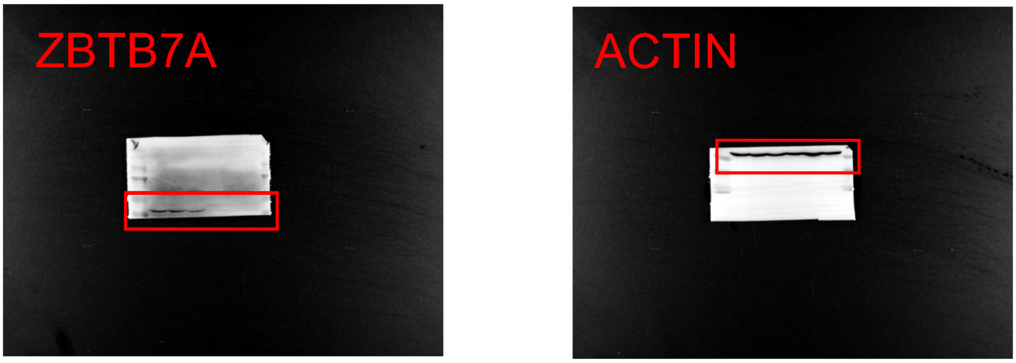


Figure 8J

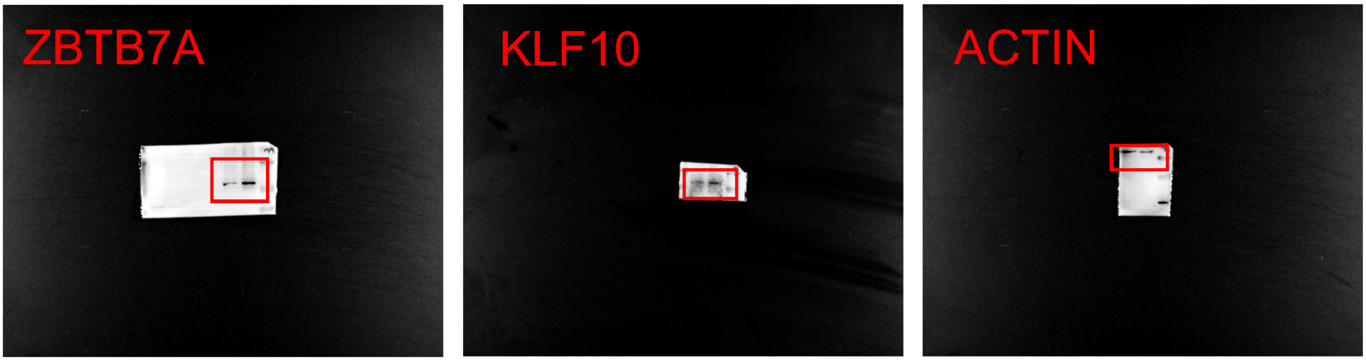

Supplementary Figure 2A

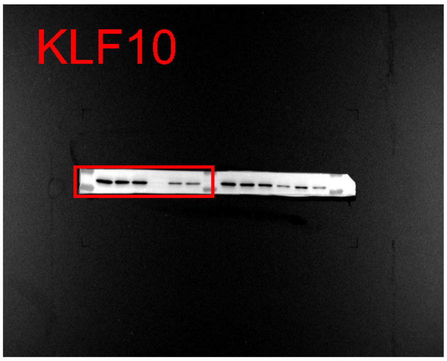


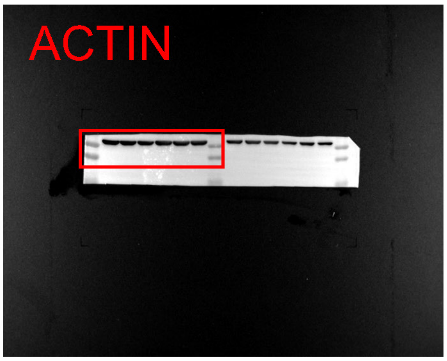


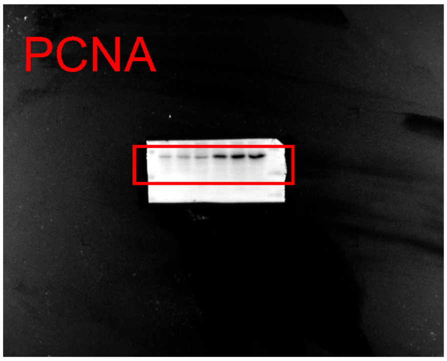


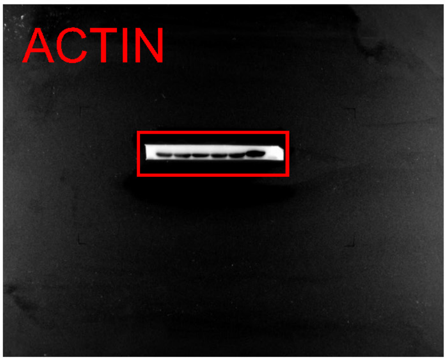


Supplementary Figure 2B

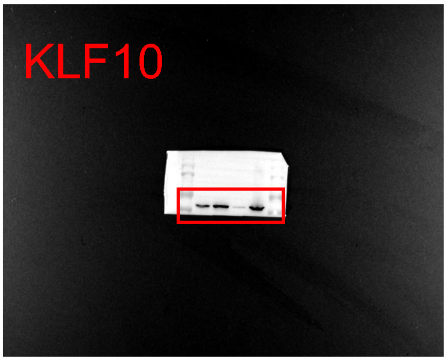


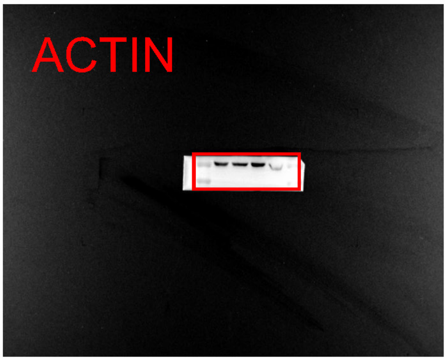

Supplementary Figure 2C

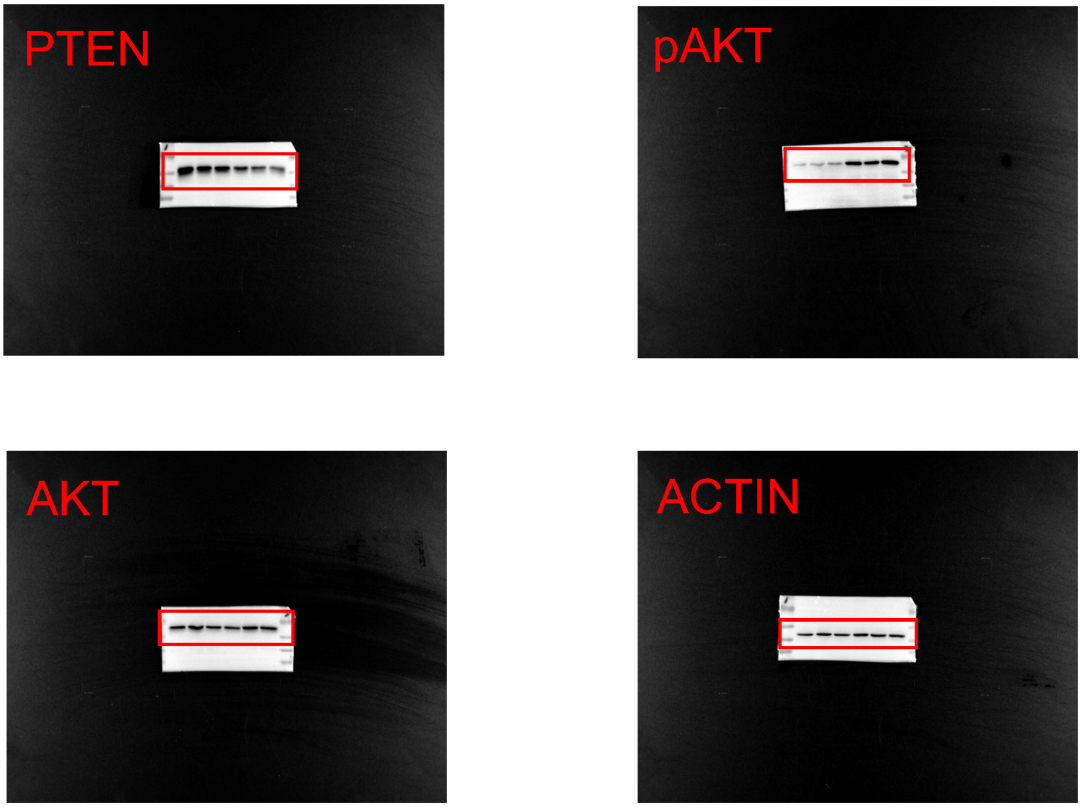


Supplementary Figure 2D

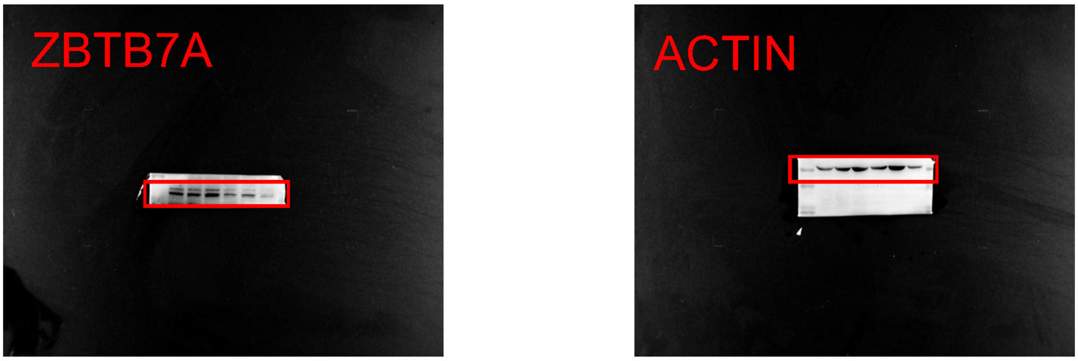

Supplementary Figure 3A

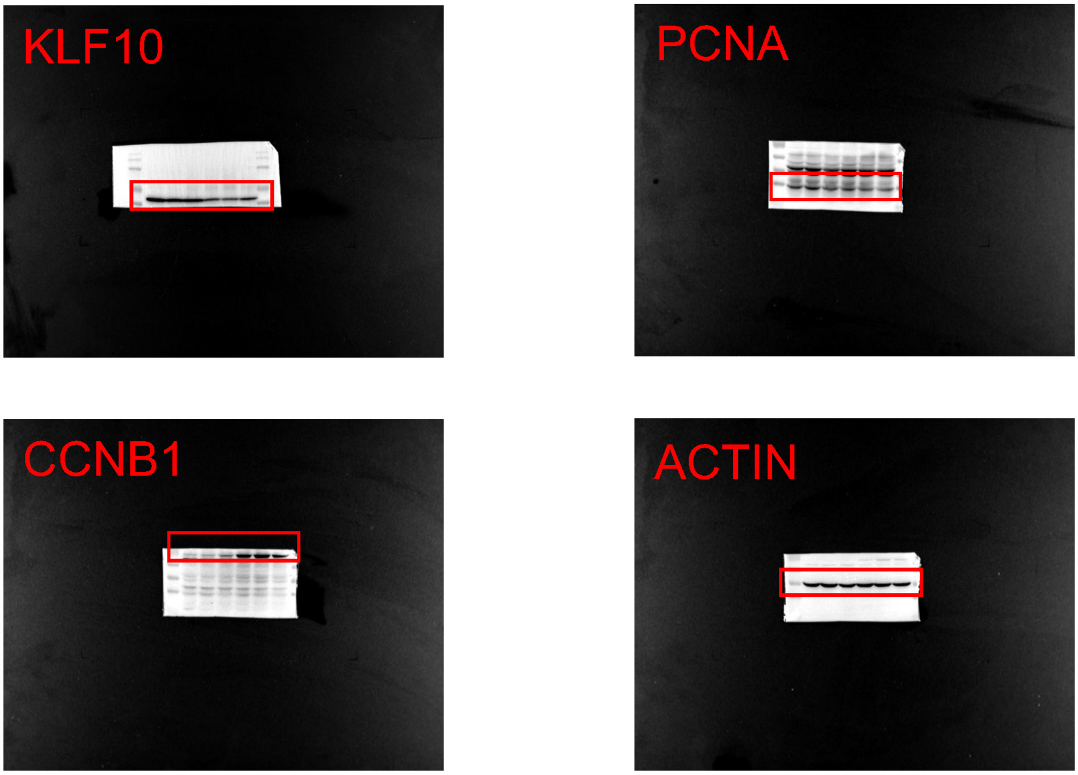


Supplementary Figure 3B

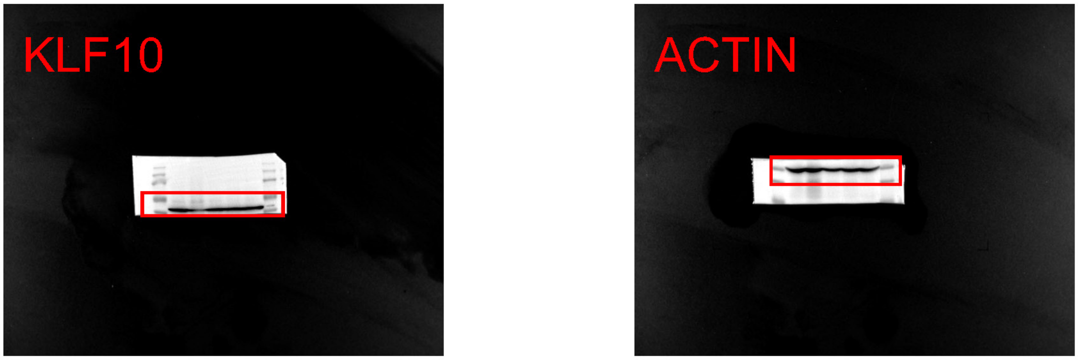

## Supplementary Figure 3C

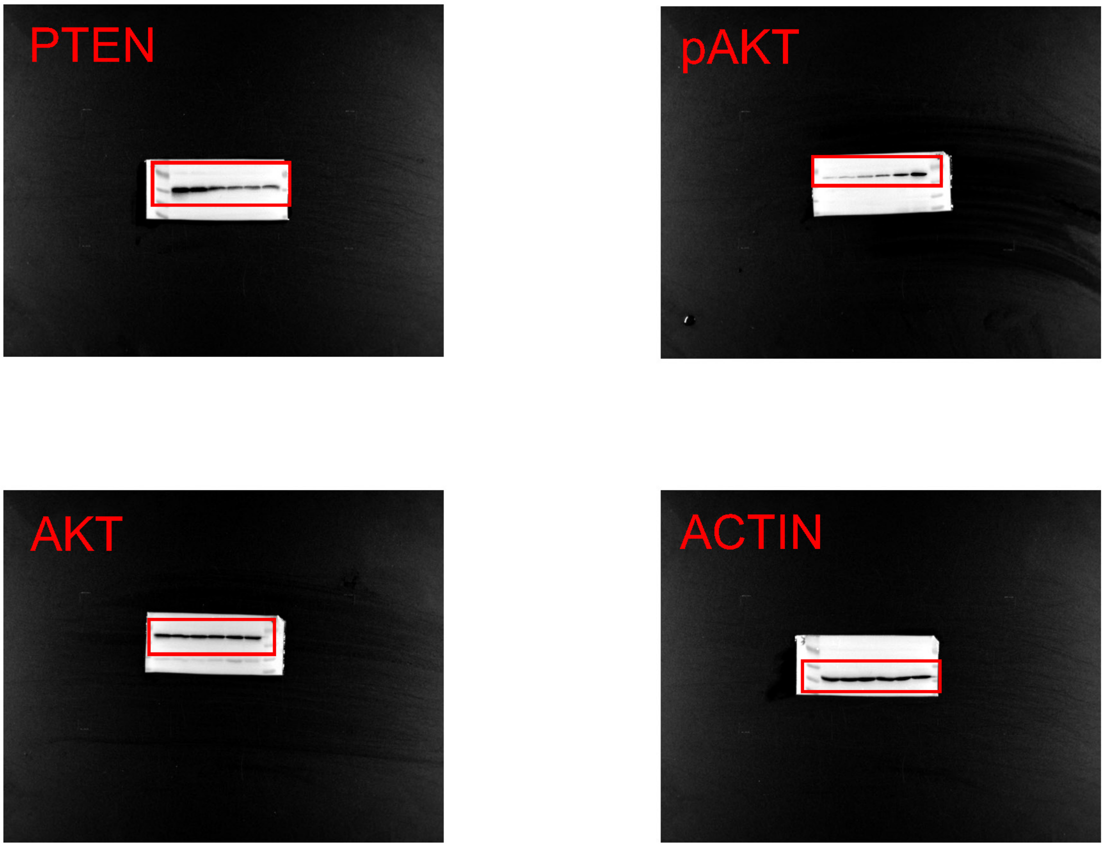


## Supplementary Figure 3D

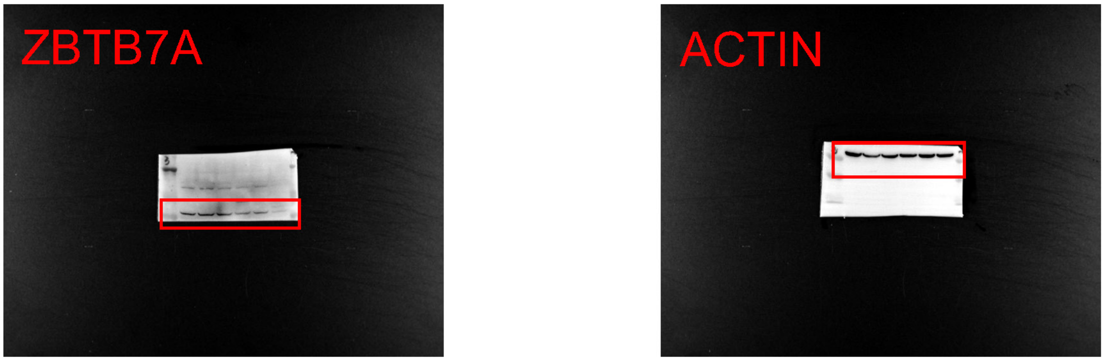

Supplement: Supplementary file 3 — Original Data File [file 41420_2023_1381_MOESM3_ESM.pdf]
